# Crossover interference and sex-specific genetic maps shape identical by descent sharing in close relatives

**DOI:** 10.1371/journal.pgen.1007979

**Published:** 2019-12-20

**Authors:** Madison Caballero, Daniel N. Seidman, Ying Qiao, Jens Sannerud, Thomas D. Dyer, Donna M. Lehman, Joanne E. Curran, Ravindranath Duggirala, John Blangero, Shai Carmi, Amy L. Williams

**Affiliations:** 1 Department of Molecular Biology and Genetics, Cornell University, Ithaca, New York, United States of America; 2 Department of Computational Biology, Cornell University, Ithaca, New York, United States of America; 3 South Texas Diabetes and Obesity Institute and Department of Human Genetics, University of Texas Rio Grande Valley School of Medicine, Brownsville, Texas, United States of America; 4 Department of Medicine, University of Texas Health Science Center at San Antonio, San Antonio, Texas, United States of America; 5 Braun School of Public Health and Community Medicine, The Hebrew University of Jerusalem, Jerusalem, Israel; Regeneron Genetics Center, UNITED STATES

## Abstract

Simulations of close relatives and identical by descent (IBD) segments are common in genetic studies, yet most past efforts have utilized sex averaged genetic maps and ignored crossover interference, thus omitting features known to affect the breakpoints of IBD segments. We developed Ped-sim, a method for simulating relatives that can utilize either sex-specific or sex averaged genetic maps and also either a model of crossover interference or the traditional Poisson model for inter-crossover distances. To characterize the impact of previously ignored mechanisms, we simulated data for all four combinations of these factors. We found that modeling crossover interference decreases the standard deviation of pairwise IBD proportions by 10.4% on average in full siblings through second cousins. By contrast, sex-specific maps increase this standard deviation by 4.2% on average, and also impact the number of segments relatives share. Most notably, using sex-specific maps, the number of segments half-siblings share is bimodal; and when combined with interference modeling, the probability that sixth cousins have non-zero IBD sharing ranges from 9.0 to 13.1%, depending on the sexes of the individuals through which they are related. We present new analytical results for the distributions of IBD segments under these models and show they match results from simulations. Finally, we compared IBD sharing rates between simulated and real relatives and find that the combination of sex-specific maps and interference modeling most accurately captures IBD rates in real data. Ped-sim is open source and available from https://github.com/williamslab/ped-sim.

## Author summary

Simulations are ubiquitous throughout statistical genetics in order to generate data with known properties, enabling tests of inference methods and analyses of real world processes in settings where experimental data are challenging to collect. Simulating genetic data for relatives in a pedigree requires the synthesis of chromosomes parents transmit to their children. These chromosomes form as a mosaic of a given parent’s two chromosomes, with the location of switches between the two parental chromosomes known as crossovers. Detailed information about crossover generation based on real data from humans now exists, including the fact that men and women have overall different rates (women produce ∼1.6 times more crossovers) and that real crossovers are subject to interference—whereby crossovers are further apart from one another than expected under a model that selects their locations randomly. Our new method, Ped-sim, can simulate pedigree data using these less commonly modeled crossover features, and we used it to evaluate the impact of sex-specific rates and interference compared to real data. These comparisons show that both factors shape the amount of DNA two relatives share, and that their inclusion in models of crossover better fit data from real relatives.

## Introduction

Inferring identical by descent (IBD) segments and estimating relatedness are classical problems in human genetics [1], with recent work motivated by the abundance of close relatives in large samples [2–6]. In order to study individuals with a known relationship, many investigators have performed simulations, both to evaluate novel methods [4–8], and to characterize the properties of IBD sharing rates among relatives [9, 10]. Additionally, direct-to-consumer genetic testing companies—now with data from several million individuals—have used simulated data to infer relationships by matching relatedness statistics from their customers to those from simulations [11, 12].

In parallel with the above, efforts to characterize crossovers, including the dynamics of crossover interference [13–15] and differences in male and female genetic maps [15–17] have yielded precise resources for realistically simulating this form of recombination. Despite this, most prior simulations and canonical models of IBD sharing between relatives [18] make use of sex averaged genetic maps and have ignored crossover interference.

Differences between male and female crossover rates were first identified decades ago [19], and modern data from families enable detection of separate male and female crossovers and therefore the inference of distinct maps [15–17]. Other commonly used but inherently sex averaged genetic maps [20] are based on population linkage disequilibrium (LD) patterns: the signal left by thousands of male and female meioses. Crossover events are also detectable as positions that switch between ancestral populations within an admixed individual’s genome, but—lacking information about which ancestor produced each crossover—the resulting maps are also sex averaged [21]. Lastly, recent work on sequencing and resolving crossovers in single oocytes [22, 23] and sperm cells [24–27] provide information on sex-specific crossover properties and can be used to construct sex-specific maps.

In turn, characterization of crossover interference, initially observed as unexpectedly low rates of double crossovers in early *Drosophila* linkage analyses [28], now includes sex-specific parameter estimates from over 18,000 human meioses [15]. During meiosis, crossovers arise as chiasmata that physically link homologous chromosomes within tetrads—four chromosome bundles consisting of two copies of each homologous chromosome. Since chiasmata link together two non-sister chromatids (i.e., homologous chromosome copies of distinct parental origin), the resulting crossover affects just two of the four gametes. To account for crossover interference, early models assumed that crossover intermediates are placed uniformly at random, but that only one of *m* intermediates resolves as a chiasma [29, 30]. The inter-chiasmata distance under this model is gamma distributed with an integer shape parameter *m* (the *χ*^2^ model). This and current models assume a uniformly random placement of chiasmata among the chromatids in a tetrad (i.e., no chromatid interference [22, 23]), corresponding to an independent probability of 1/2 for a gamete to contain any crossover. Later work found that inter-chiasmata distances are better fit by a gamma distribution with a fractional shape parameter [13] (the gamma model). Building on this, Housworth and Stahl found improved fits to human inter-crossover distances using a mixture (two-pathway) model that, in addition to the gamma model, also includes some fraction of events that escape interference [14].

Here, we employ empirical human genetic maps and interference estimates to analyze the effects of crossover modeling on IBD distributions between close relatives. Specifically, we simulated several types of relatives using either sex-specific or sex averaged crossover genetic maps [16], and either incorporating crossover interference (under the Housworth-Stahl model) [14, 15] or using a non-interference (i.e., Poisson) model. While mean IBD sharing rates are unaffected by these factors, the variance in IBD sharing proportion differs substantially between them, impacting relationship classification metrics (particularly between close relatives) and estimates of the time since admixture for very recently admixed individuals. Furthermore, by analytically solving a theoretical renewal process model, we show that crossover interference impacts the distribution of IBD segment lengths, and we confirm these results using simulations.

We conducted all simulations for this study using Ped-sim, an open source method we developed that simulates relatives under any of the four combinations of genetic map type and inter-crossover distance model (Methods). Ped-sim has functionality related to IBDsim [31], but the latter uses a *χ*^2^ interference model with fixed parameters, is less scalable than Ped-sim (Results), and does not produce genetic data.

To determine which crossover model best fits data from real relatives, we leveraged genotypes from the San Antonio Mexican American Family Studies (SAMAFS) [32–34], a dataset comprising roughly 2,500 samples in dozens of pedigrees. With thousands of close relative pairs, these data enable precise estimates of IBD summary statistics. We also leveraged IBD sharing rates from 20,240 full sibling pairs analyzed by Hemani et al. [35]. These analyses show that use of sex-specific genetic maps and interference modeling provide overall better fits to IBD sharing summary statistics in these real relatives than do other crossover models.

## Results

To investigate the effect of sex-specific maps and crossover interference on IBD sharing between relatives, we used Ped-sim to simulate 10,000 pairs of relatives for several relationship types and each of four crossover models. Ped-sim can produce genetic data for relatives given input haplotypes, but the analyses we present leverage exact IBD segments as detected through internally tracked haplotype segments (Methods). These segments arise by (simulated) descent from the chromosomes of founders—i.e., pedigree members whose ancestors Ped-sim does not model.

Comparing IBD sharing between simulated and real relatives is complicated by the fact that deeper, “background” relatedness from cryptic common ancestors can exist between real samples [36]. This may inflate the relatedness between real samples above that implied by the more recent common ancestors we focus on. Additionally, population-based IBD segment inference procedures are subject to both false positive and false negative signals, whereas simulated data perfectly capture the IBD regions generated under a given model.

We used two approaches to detect IBD segments in the SAMAFS data: one family-based and applied to full siblings—a strategy that avoids most issues of background relatedness, as described next—and a population-based detector for other relatives [37]. The population-based IBD estimates require adjustment for background relatedness and false signals, so we mean-shifted these estimates to match theoretical expectations in each relationship class. Furthermore, while we analyze the standard deviation of IBD sharing rates in full siblings, to limit the impact of outliers in quantifying the corresponding variance terms within non-sibling relatives, we focus on the quartiles of the mean-shifted IBD sharing fractions. (Standard deviations derive from squared deviations from the mean, so outliers have a stronger influence on that statistic than on the rank-ordered quartiles).

To calculate the full sibling IBD sharing proportions, we applied a family-based phasing method [38] to the SAMAFS nuclear families that have data for at least three children and both parents. Likewise, we leverage IBD estimates from 20,240 full sibling pairs that Hemani et al. inferred using a family-based algorithm (Hemani20k) [35, 39]. These family-based IBD detection methods work by inferring haplotype transmissions from parents to children and locating regions where a pair of children co-inherit the same parental haplotype. That is, the siblings’ IBD status is with respect to the parents’ four haplotypes, not the alleles the siblings share, so the detected IBD segments are only those inferred to coalesce in the parents (not older, cryptic relatives). Moreover, family-based phasing models are extremely accurate and are considered to be the gold standard approach [40], leading to IBD sharing estimates that deviate little from the truth (95% confidence interval of deviation [−1.73 × 10^−3^, 2.25 × 10^−3^] in simulated three child families; S1 Fig). Notably, background IBD sharing between the two parents has little impact on the phasing quality due to the depth of information contained in a nuclear family, including the long-range linkage of haplotypes. Additionally, a run of homozygosity (ROH) in a parent, though inhibiting precise localization of crossovers, also rarely confounds IBD detection due again to linkage. (In general, the majority of children will have inherited a non-recombined haplotype across the ROH interval, enabling phasing of sites surrounding the ROH.) Given these factors, we consider the IBD sharing values inferred for the real full siblings as comparable to the corresponding simulated data quantities, and we do not adjust them.

The IBD proportions quoted hereafter are fractions of the diploid genome two samples share, and we calculated these as half the fraction of the genome the two share IBD1 plus the IBD2 fraction (Methods), where these IBD1/IBD2 regions correspond to locations the pair shares on one or two haplotypes, respectively. Below, we abbreviate sex-specific and sex averaged as SS and SA, respectively, and refer to the four crossover models we used with Ped-sim as: SS+intf for sex-specific genetic map with interference; SS+Poiss for sex-specific map, Poisson event distribution (i.e., no interference); SA+intf for sex averaged genetic map with interference; and SA+Poiss for sex averaged map, Poisson event distribution.

### Sex-specific maps and interference oppositely affect the variance in IBD sharing proportion

**Fig 1:**
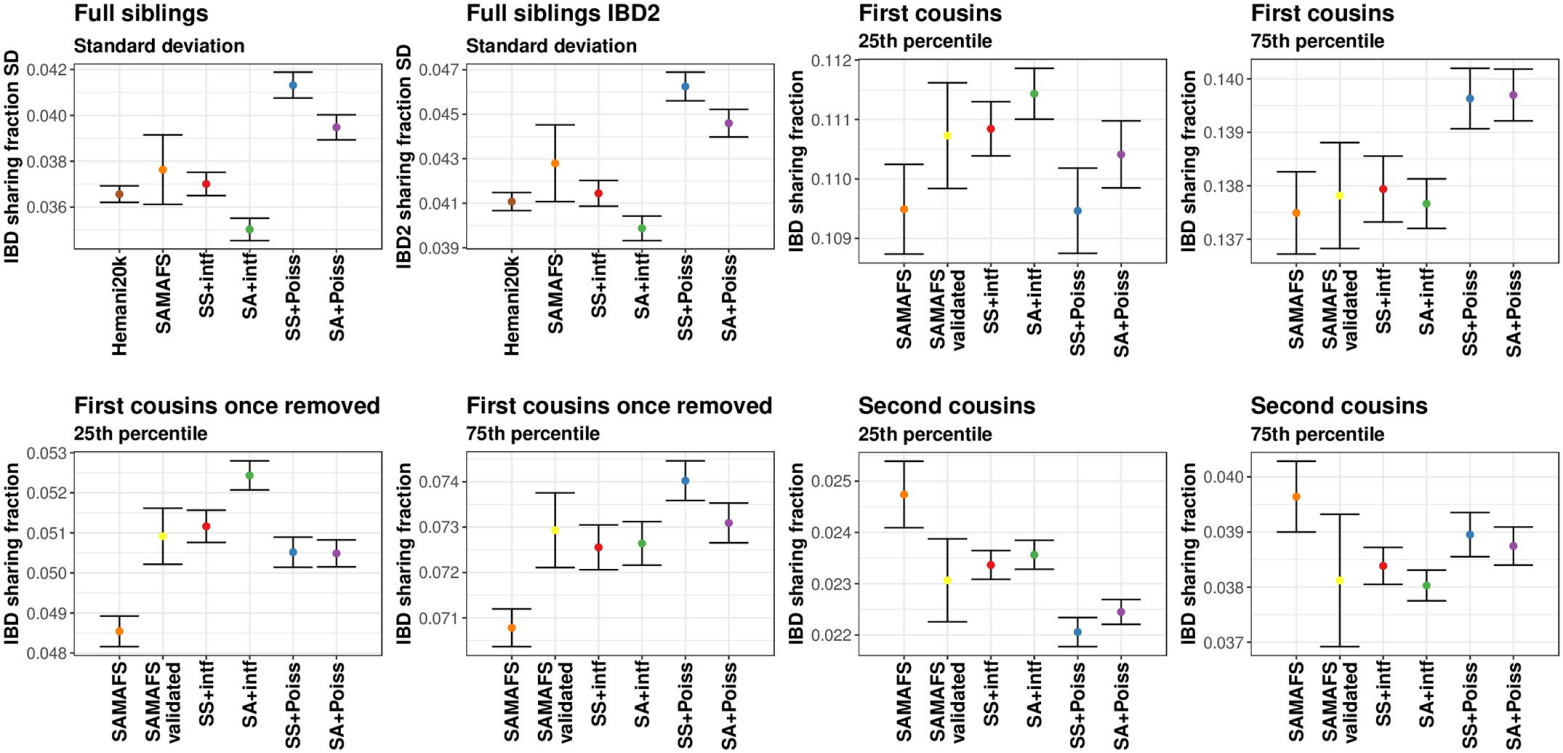
IBD sharing fraction standard deviations in full siblings and 25th and 75th percentiles in first through second cousins from real and simulated data. Points are from the SAMAFS, SAMAFS-validated subset (except full siblings), Hemani20k set (only full siblings), and the simulation models. The latter are labeled using abbreviations given in the main text. The SAMAFS and SAMAFS-validated 25th and 75th percentiles are from values mean-shifted to match expectations. Bars indicate 95% confidence interval (±1.96 standard errors) as calculated from 1,000 bootstrap samples. Standard deviations for first through second cousins and 25th and 75th percentiles for full siblings are in S2 Fig, and further statistics are in S3 Fig. SD indicates standard deviation.

We simulated full siblings, first cousins, first cousins once removed, and second cousins under all four crossover models. For all relative types, use of SS genetic maps increases the variance in IBD proportion compared to the SA map, though the effect is somewhat limited. In particular, averaged among these relationships, the standard deviation increases by 3.6% under the Poisson crossover localization model and 4.7% under the interference model (Fig 1, S2 Fig). SS maps have similar effects on the size of the interquartile range, increasing this span by an average of 3.9% under the Poisson model and 5.3% in the presence of crossover interference. These small differences in IBD sharing statistics correspond to distributions of IBD rates for SS and SA maps that are difficult to distinguish visually (S4 Fig).

**Fig 2:**
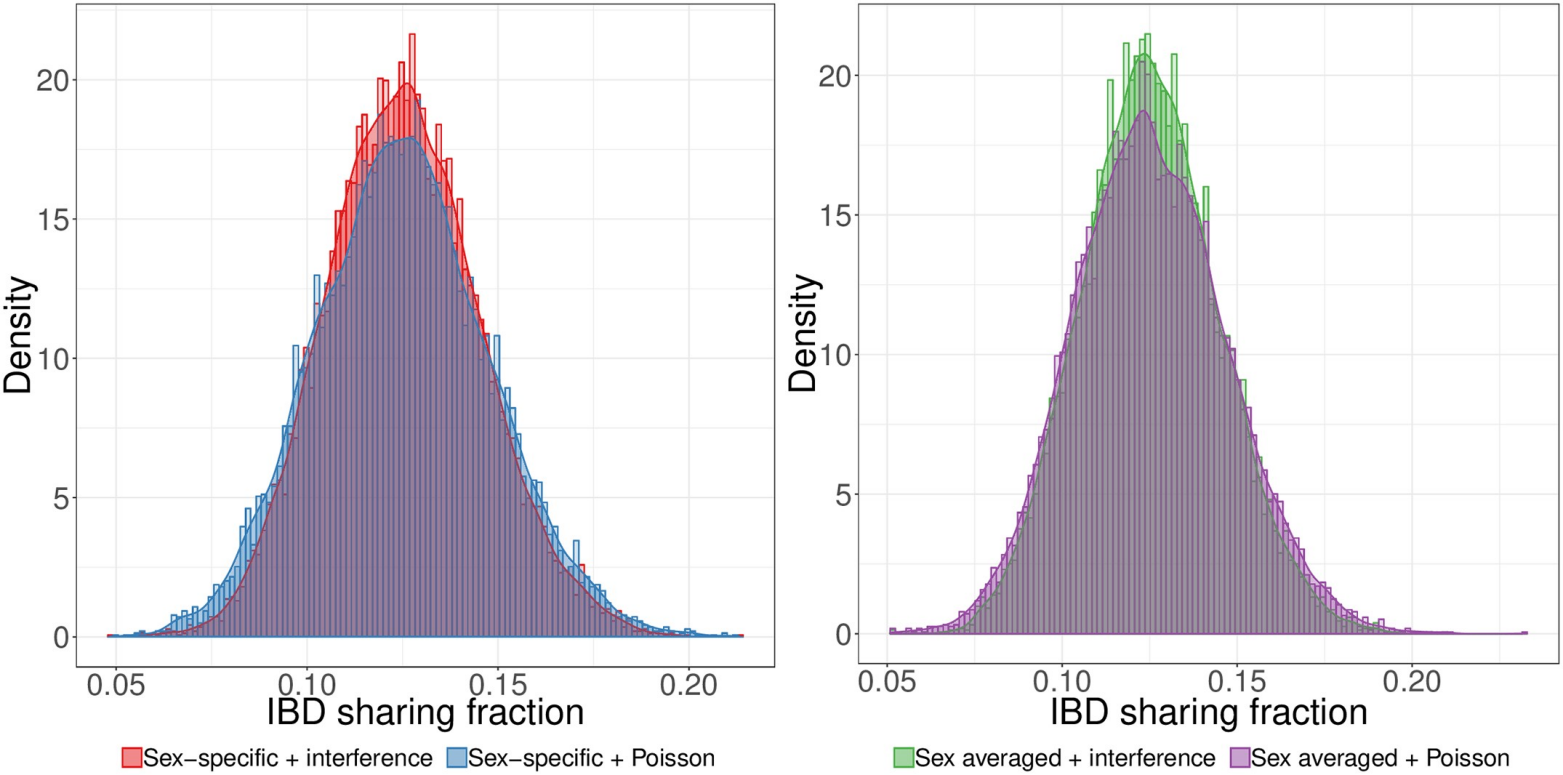
First cousins simulated with crossover interference have a distribution of IBD sharing proportion more concentrated near the mean than those simulated using a Poisson model. Interference decreases the variance in IBD sharing both when using sex-specific (left) and sex averaged (right) genetic maps.

By contrast, crossover interference has a strong effect on the variance in IBD sharing fraction, decreasing the standard deviation compared to the Poisson model by 10.0% when simulating with SS maps and 10.9% using the SA map (averaged over all relationships we considered; Fig 1, S2 Fig). Furthermore, interference tightens the range between the 25th and 75th percentiles by 9.8% when using SS maps and 11.0% using the SA map. With decreased variances of these magnitudes, the distributions of IBD proportions for relatives simulated under interference are noticeably more peaked near the mean, with smaller tails (Fig 2). These results highlight the importance of including interference when simulating relatives, and hint that distantly related samples may have non-zero IBD sharing more frequently when simulated under interference—a feature we analyze below (see “Rates of sharing at least one IBD segment among distant relatives”).

### Simulations including sex-specific maps and interference best fit data from real relatives

Given the differences in the distribution of IBD proportions observed by varying the combination of map type and crossover interference among simulated relatives, we sought to understand which scenario best matches real human data. We first examined IBD sharing between pairs of full siblings in the SAMAFS and Hemani20k data, which have mean IBD proportions of 0.501 and 0.502, respectively, in line with expectations (S3 and S5 Figs). Overall, the SS+intf model produces the best fit to the standard deviations from real data, being the only model within one standard error (0.76 units) of the SAMAFS estimate, and 1.03 standard errors from the Hemani20k value (Fig 1, S6 and S7 Figs). This contrasts with the traditional SA+Poiss model, which is 2.3 standard errors from SAMAFS, and 9.3 from Hemani20k. The SA+intf and SS+Poiss models are also discrepant, with both more than 3.2 standard errors from SAMAFS, and 3.4 standard errors from Hemani20k.

The mean IBD2 sharing rate in the SAMAFS full siblings is 0.250, as expected, and the corresponding value in Hemani20k is 0.251 (S3 Fig). The standard deviation of IBD2 sharing under the SS+intf model is 1.4 standard errors from that of SAMAFS, and only 1.03 standard errors from the Hemani20k value. Again, these deviations are the smallest of all the models we considered (Fig 1). The traditional SA+Poiss model is the next closest to SAMAFS at a distance of 1.9 standard errors, but deviates meaningfully from Hemani20k at 9.3 standard errors away. The SA+intf IBD2 standard deviation is 3.0 and 3.4 standard errors from the SAMAFS and Hemani20k quantities, respectively, and, as in the full IBD proportion, SS+Poiss deviates the most from the real data, being 3.6 standard errors from SAMAFS, and 13.3 from Hemani20k.

Turning to relationships more distant than full siblings, we focus on the interquartile IBD sharing rates compared to mean-shifted SAMAFS values. Additionally, we analyzed a subset of SAMAFS samples for whom data for all first degree relatives that connect them are available, and where we confirmed these first degree relationships (S8A Fig, Methods). This subset should be free of any mislabeled relatives, and we refer to it as SAMAFS-validated.

As in the full sibling analyses, use of SS genetic maps and crossover interference modeling provides a good fit to the real data across all these more distant relationship types. In first cousins, the 25th and 75th percentile IBD proportions under the SS+intf model are 0.111 and 0.138—the same as in the SAMAFS-validated data (Fig 1)—while the corresponding percentiles under SA+Poiss are 0.110 and 0.140, with the latter value 3.3 standard errors from SAMAFS-validated. For first cousins once removed and second cousins, the SS+intf 25th and 75th percentile values are within one standard error of SAMAFS-validated in all cases, while the three other models deviate by more than one standard error for at least one of the two quartiles in both relationships.

As another line evidence that IBD sharing in SS+intf most closely mirrors that of real data, we inferred degrees of relatedness for the simulated and SAMAFS relatives. This inference maps the kinship coefficient of each pair to a degree of relatedness using the same kinship ranges as in KING [41] (Methods). S9 Fig plots the percentage of samples inferred as their true degree of relatedness in the SAMAFS and simulated pairs. For all four relationship types, the model with percentages nearest to that of SAMAFS-validated is SS+intf. In fact, SS+intf is within one standard error of the SAMAFS-validated percentage for all four relationship types, whereas SA+Poiss and SS+Poiss are >3.0 standard errors from SAMAFS-validated for all but full siblings. The SA+intf model is less than one standard error away from SAMAFS-validated for all but first cousins once removed, where it deviates by 2.4 standard errors.

### Rates of sharing at least one IBD segment among distant relatives

**Fig 3:**
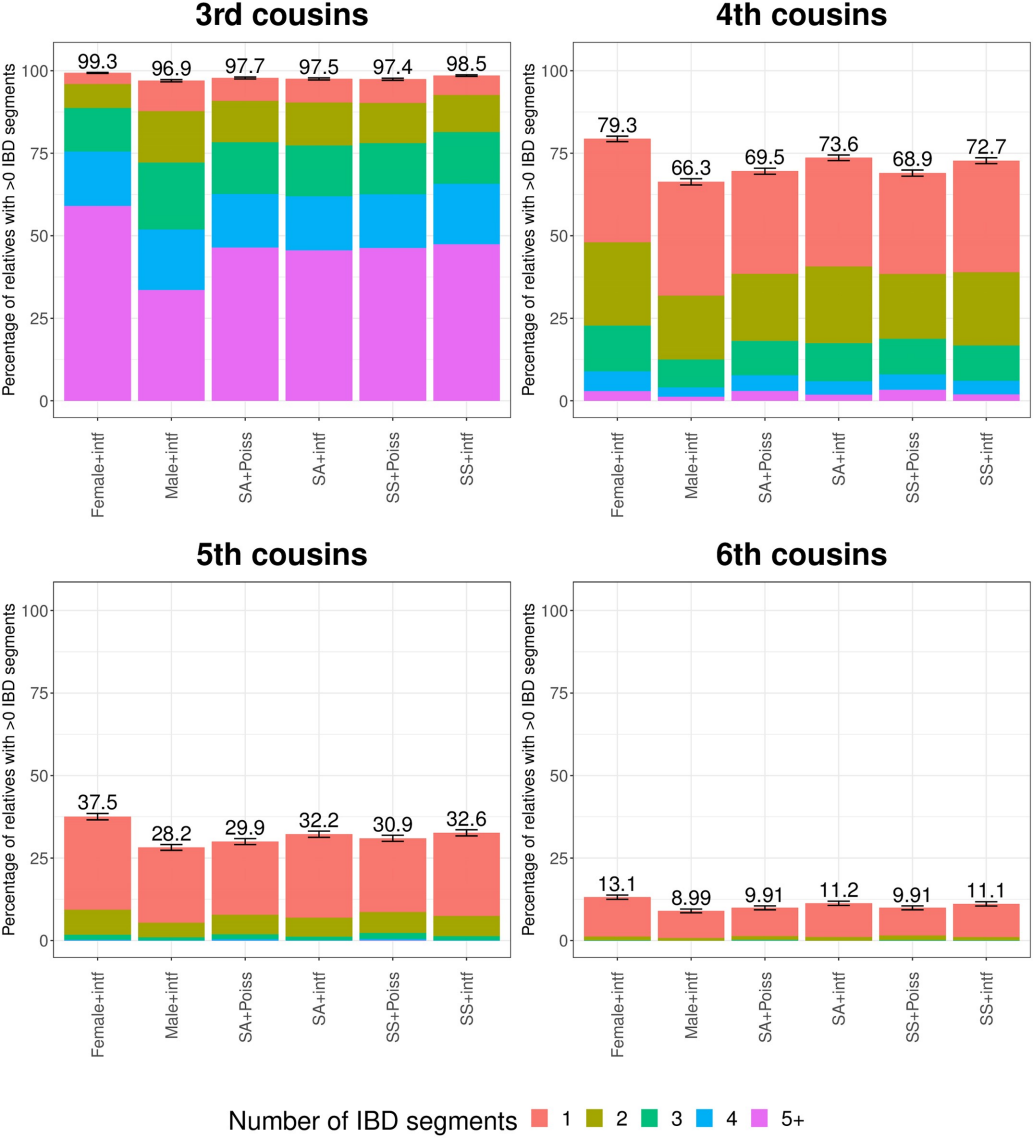
Number of IBD segments that simulated third through sixth cousins share under various modeling scenarios. More distant relatives have reduced rates of sharing one or more IBD regions. Percentages above each bar indicate the fraction of simulated relatives (of 10,000 for each scenario) that have at least one segment shared. Female+intf are from simulations using sex-specific maps and interference but where the pairs are related through only female non-founders, with a male and female couple as founder common ancestors (S8B Fig). Male+intf pairs are the same as Female+intf but with the non-founders being only male instead of female. Error bars are the 95% confidence interval (±1.96 standard errors) of the percentage of relatives that share at least one IBD segment based on 1,000 bootstrap samples. Error on internal bar segment counts are in S10 Fig.

Random assortment during meiosis commonly leads to a loss of IBD segments such that distant relatives may not share any IBD regions with each other despite having a genealogical relationship. Given the fit of the crossover model that incorporates SS maps and interference, we set out to examine the distribution of the number of IBD segments shared among full and half-siblings and first through sixth cousins. For close relatives, including full and half-siblings, and first and second cousins, all simulated pairs share at least one IBD segment with each other regardless of the crossover model. However, some proportion of third through sixth cousins share no IBD segments of any size (Fig 3). Specifically, in the SS+intf simulation, 1.5% of third cousins share no IBD regions, and this percentage increases to 27.3%, 67.4%, and 88.9% of fourth, fifth, and sixth cousins, respectively. For the 1,112 (of 10,000) sixth cousins that do share IBD segments, the average total length is 7.6 centiMorgans (cM). Unsurprisingly, most sixth cousin pairs retain only one IBD segment with very few (107/1,112) pairs sharing more than one segment (Fig 3). The total IBD length varies substantially among sixth cousins, with the top 25% of pairs that have IBD regions sharing a total of at least 10.2 cM and a maximum of 53.4 cM. Thus sixth cousins with rare extremes of IBD sharing have total shared lengths more typical of third and fourth cousins.

As already noted, crossover interference leads to a more concentrated distribution of IBD sharing rates (e.g., Fig 2). Interference also leads to a slightly larger fraction of distant relatives that share IBD segments. For example, 32.7% of fifth cousins share one or more IBD segments under the SS+intf model compared to only 30.0% under SA+Poiss.

### Sex-specific maps impact the number of IBD segments relatives share

While SS maps have a smaller effect than interference on the variance in IBD sharing proportion between two relatives, they do impact the number of segments relatives share. Specifically, females produce an average of 1.57 times more autosomal crossover events per meiosis than males [16]. With such differences, females should transmit a larger number of IBD segments that are on average smaller compared to transmissions from males. This is because, without a crossover event, the probability of transmitting an IBD segment is 50%. On the other hand, when a newly generated crossover occurs within an IBD region, transmission of some portion of the IBD region (on one side or the other of the crossover) is guaranteed.

To more fully investigate the impact of SS genetic maps, we used the SS+intf model to simulate third through sixth cousins where the non-founder ancestors through whom they are related are either all female or all male (with the shared founder grandparents being a male and female couple; S8B Fig). When related primarily through females, third through sixth cousins are much more likely to have non-zero IBD sharing than those related primarily through males. The differences are quite extreme with respectively 2.5%, 19.6%, 33.1%, and 46.2% more (in relative terms) third, fourth, fifth, and sixth female-lineage cousin pairs sharing at least one IBD region compared to the analogous male-lineage cousins (Fig 3). Consistent with intuition, the IBD regions in female-descent cousins are smaller on average than those in male-descent cousins. For example, female-lineage fifth cousins with IBD regions share an average of 1.3 segments with a mean total length of 9.0 cM compared to the male-lineage averages of 1.2 segments and total length 11.9 cM.

**Fig 4:**
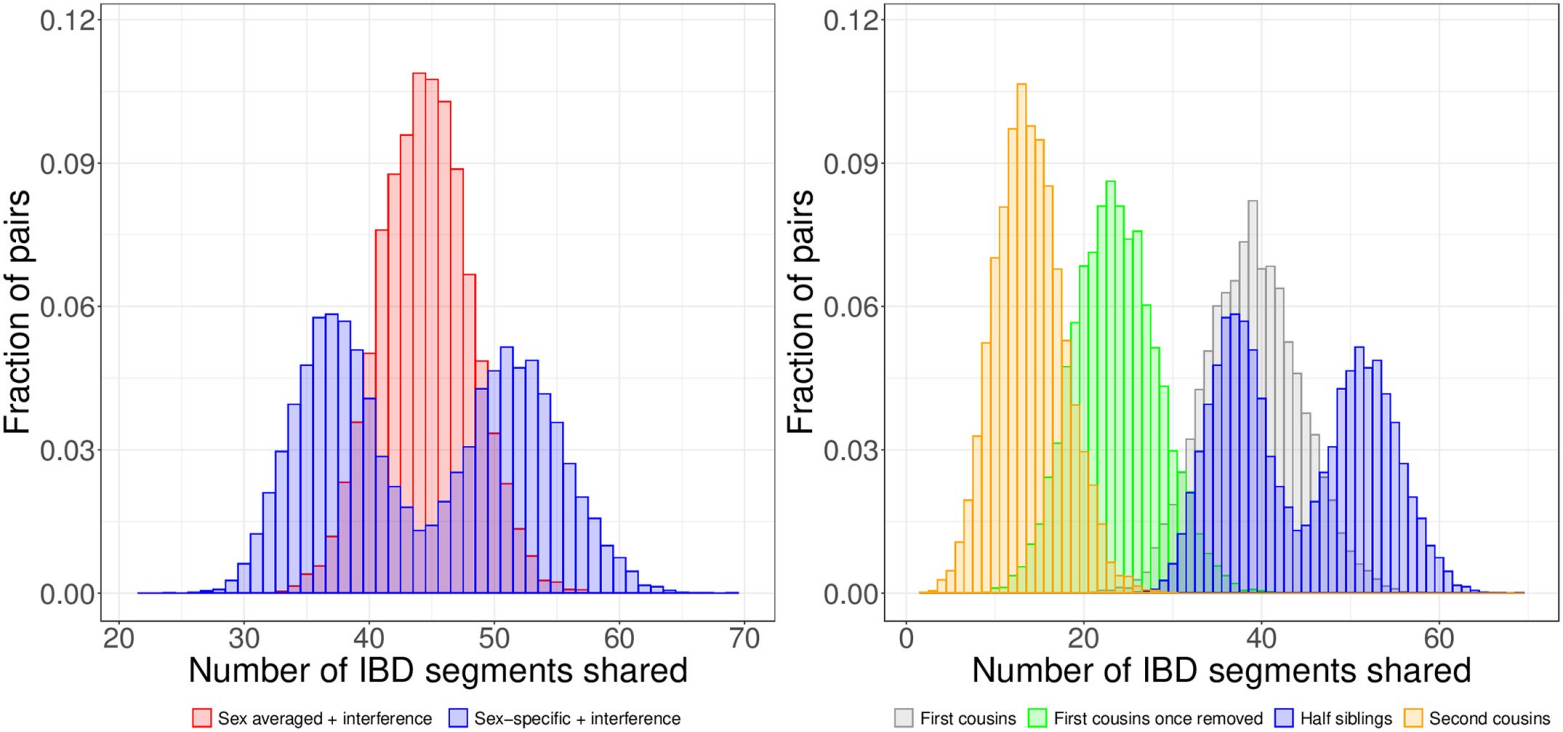
Sex-specific maps impact the number of segments half-siblings share. Number of IBD segments half-siblings share when simulated with sex averaged maps compared to sex-specific maps have very different shapes, with sex-specific maps producing a bimodal distribution (left). Half-sibling segment counts in the context of other relative types where we simulated all relatives under sex-specific maps (right). The lower mode of half-sibling segment counts—which corresponds to IBD sharing between paternal half-siblings (S11A Fig)—is below that of first cousins. The distributions are based on 10,000 pairs simulated under interference for all relationship types.

These differences in male and female maps impact IBD sharing between close relatives as well, with especially noticeable effects in half-siblings. In particular, maternal half-siblings share on average 1.4 times as many IBD segments as paternal half-siblings (mean segment numbers 51.9 and 37.1, respectively). The effect is substantial enough to produce a bimodal distribution, with little overlap between the two types of half-siblings (Fig 4; S11A Fig). Although less distinct than segment counts from simulations, the SAMAFS half-siblings also have a bimodal distribution that corresponds with the sex of the common parent (S11B Fig; Methods). Notably, the mean segment count in simulated paternal half-siblings is less than that of first cousins with randomly assigned parent sex (who share a mean of 39.0 segments; Fig 4). However, the segments paternal half-siblings share are more than twice as long as those of first cousins, with an average length of 45.1 cM compared to 21.5 cM in first cousins.

### Inferring degrees of relatedness from simulation summary statistics

Given the popularity of genetic testing companies and their work to infer relatedness by matching real data summary statistics to corresponding values from simulations [11, 12], we sought to understand the impact the simulation model has on this inference. In particular, we examined classification rates and relationship probability calibrations in kernel density estimation models (KDEs) trained under the four crossover scenarios (Methods). We applied these KDEs to data simulated under SS+intf, which more precisely captures real data relatedness statistics compared to the other models (Fig 1, S9 Fig).

As shown in S12 Fig, the sensitivity and specificity of the KDEs are fairly similar among the four types of training data. However, the classifier trained using SA+Poiss data has lower specificity overall and lower sensitivity for fifth and sixth degree relationships. The latter SA+Poiss specificity rates are 0.031 and 0.020 less, respectively, than the SS+intf classifier (*P* = 7.7 × 10^−7^ and *P* = 7.5 × 10^−4^, respectively, paired difference *t*-test). The calibration curves also reveal differences in performance among the training data types (S13 Fig). Under this metric, training with data subject to interference meaningfully improves the probability calibrations for second and third degree relatives compared to training with SA+Poiss data. These results suggest that, in applications that use relationship probabilities, interference modeling (including via simulations) may be beneficial for analyses of close relatives.

### Estimates of time since admixture vary by simulation model for recently admixed samples

We sought to characterize the impact of the four crossover models on estimates of the time since admixture using single admixed samples. For this purpose, we simulated one admixed haplotype per chromosome in a set of individuals, with the onset of admixture *T* generations ago, and all ancestor couples in that generation including one member of each of two populations (S14 Fig). We used the resulting local ancestry segments to estimate the time since admixture by fitting an exponential rate to all segments from the two ancestral groups in each admixed sample (Methods).

**Fig 5:**
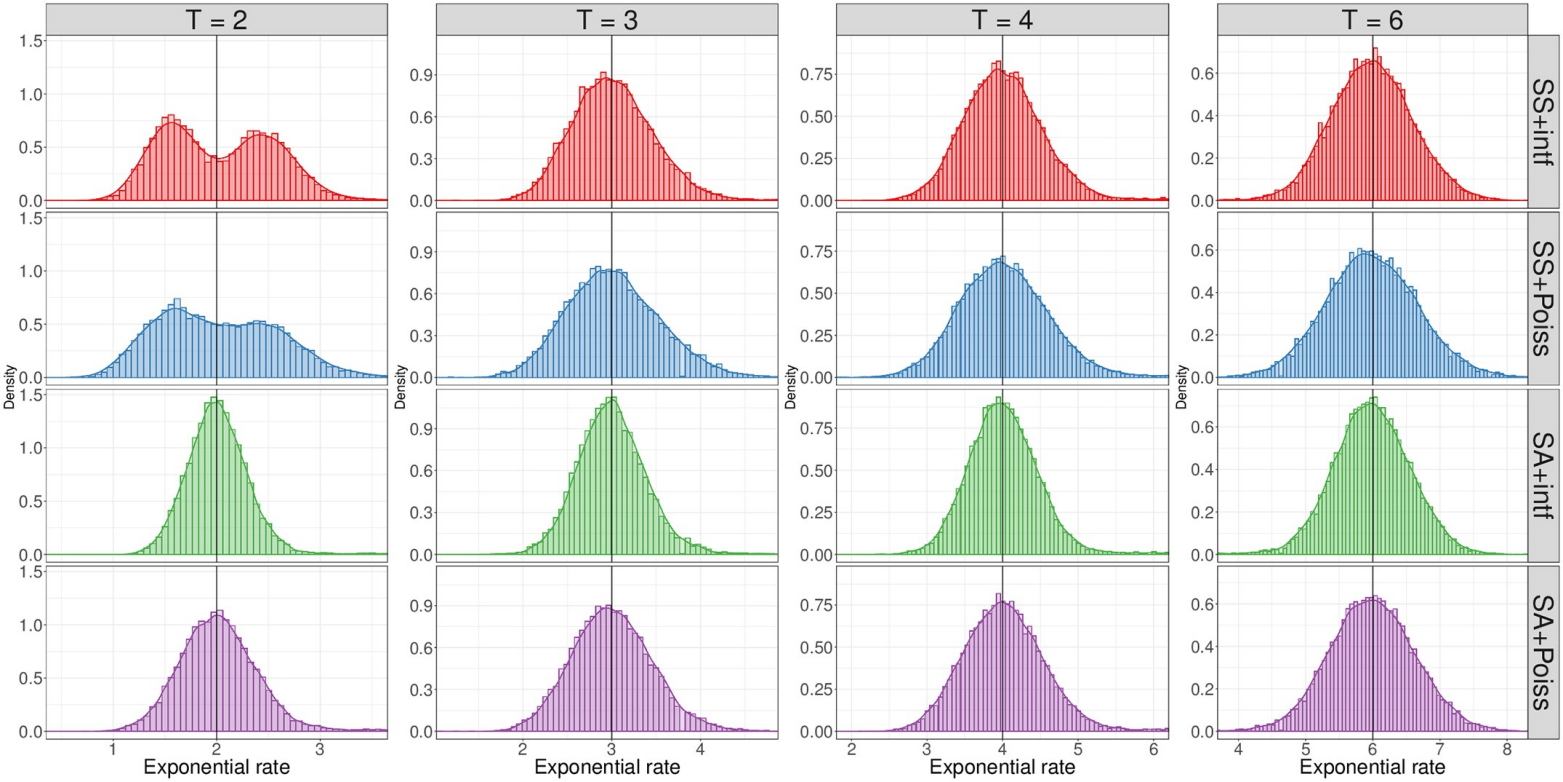
Distributions of estimated time since admixture based on one admixed sample. Histograms show estimated rates from 15,000 individuals simulated under each crossover model. Estimates are rate fits of an exponential distribution accounting for finite chromosomes (Methods). Horizontal lines indicate the true *T*.

Fig 5 plots estimates of *T* from each of 15,000 simulated admixed samples where *T* = 2, 3, 4, 6. Here, crossover interference has a noticeable effect on the distribution, leading to a reduction in the standard deviation of the estimated *T* of 11.4% compared to the Poisson model (averaged over both map types and all *T*). This effect remains consistent as *T* increases, with an 10.2% decrease in standard deviation at *T* = 2 (grandparents-grandchild) and 11.8% at *T* = 6 (fourth great-grandparents-grandchild). Additionally, the variance under SS maps is much higher than under the SA map, with standard deviations 13.4% larger under the SS maps (again averaged across interference parameters and all *T*). The impact of SS maps is greatest for small *T*, with a 23.4% larger standard deviation for *T* = 2 compared to 6.8% for *T* = 6. The differences between the SS and SA models are higher for small numbers of meioses because the probability of all meioses being in only one sex is highest for small *T*. As the number of meioses grows, a greater fraction of the samples will have closer to equal numbers of male and female meioses, and so the sharing patterns will be more similar to those that arise from an SA map.

Comparing SS+intf to the traditional SA+Poiss model shows that the effects of interference and map type in some ways cancel each other except when *T* is small. Indeed, these distributions have more similar standard deviations than the comparisons described above, with only a 6.0% lower standard deviation for SS+intf compared to SA+Poiss when *T* = 6. This indicates that, except when admixture is quite recent or for fine grained analyses, the SS+intf model produces local ancestry distributions similar to that of the SA+Poiss model. Of note, most analyses of local ancestry segments use data from many haplotypes, which should produce estimated times with overall reduced variance compared to these analyses.

### The effects of interference and sex-specific maps on IBD segment lengths

To gain insight into the effect of crossover interference and SS maps on IBD segment lengths, we analytically obtained the distribution of these lengths under the SA+intf and SS+Poiss models.

In the Housworth-Stahl two-pathway model [14], the proportion of crossovers that escape interference (are “unregulated”, or distributed according to a Poisson process) is denoted as *p*. The remaining (“regulated”) crossovers are independently generated by first drawing the positions of chiasmata as a stationary renewal process [42] along the chromosome, with gamma distributed inter-chiasma distances (in Morgans) with shape *ν* and rate 2*ν*(1 − *p*) (Methods). Each chiasma becomes a crossover in the gamete being modeled with probability 1/2. Here we consider IBD segments shared by individuals with a common ancestor *T* generations ago, or separated by 2*T* meioses.

In Methods, we show that the density of *x*, the length (in Morgans) of IBD segments subject to interference and under an SA map, is

$$\begin{array}{c} \phi (x)=e^{-2pTx}{\left[{\tilde{G}}_{\text{reg}}\left(x\right)\right]}^{2T-1}[4pTg_{\text{reg}}\left(x\right)+\frac{\left(2T-1\right)g_{\text{reg}}^{2}\left(x\right)}{{\tilde{G}}_{\text{reg}}\left(x\right)}+\left(1-p\right)f_{\text{reg}}\left(x\right)+2p^{2}T{\tilde{G}}_{\text{reg}}\left(x\right)], \end{array}$$ (1)

where

$$\begin{array}{c} f_{\text{reg}}\left(x\right)=\sum_{k=1}^{\infty }\frac{2^{-k}x^{k\nu -1}e^{-2(1-p)\nu x}{\left[2\left(1-p\right)\nu \right]}^{k\nu }}{\Gamma (k\nu )} \end{array}$$

is the probability density of the distance between regulated crossovers,

$$\begin{array}{c} g_{\text{reg}}\left(x\right)=\left(1-p\right)\sum_{k=1}^{\infty }2^{-k}\frac{\Gamma [k\nu ,2(1-p)\nu x]}{\Gamma (k\nu )} \end{array}$$

is the probability density of the distance between a random site and the next regulated crossover, and

$$\begin{array}{c} {\tilde{G}}_{\text{reg}}\left(x\right)=\int_{x}^{\infty }g_{\text{reg}}\left(y\right)dy \end{array}$$

is one minus the cumulative distribution of *g*_reg_(*x*).

The expressions above are valid for infinitely-long chromosomes. We further show in Methods how to modify Eq (1) for the case of a finite chromosome (Eq (16)).

**Fig 6:**
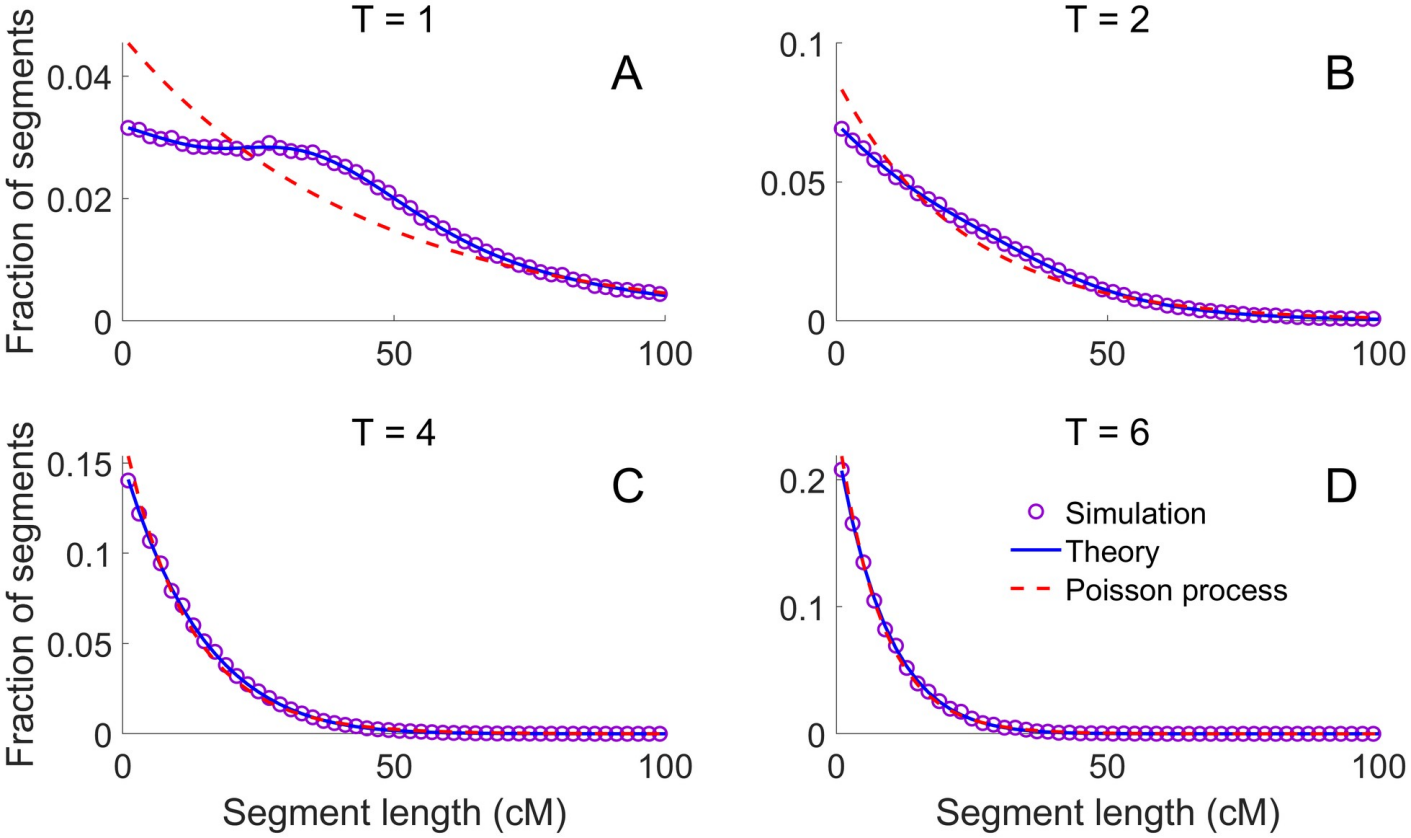
The effect of crossover interference on IBD segment lengths. We used Ped-sim to simulate half-cousins with a common ancestor *T* = 1, 2, 4, 6 generations ago (panels A-D, respectively) under the SA+intf model, extracting IBD segment lengths for chromosome 1. Each panel shows the simulated distribution of IBD segment lengths (over 10^5^ pairs for *T* = 1, 2 and 10^6^ pairs otherwise; purple circles), the theory from Eq (1) (blue lines; including the finite-chromosome correction of Eq (16)), and the expectation based on a Poisson process (red dashed lines; Eq (17)).

To confirm these results, we used Ped-sim to simulate IBD sharing under the SA+intf model for chromosome 1. The simulated distribution of the IBD segment lengths is shown in Fig 6 for half-cousins with a common ancestor *T* = 1, 2, 4, 6 generations ago (where *T* = 1 corresponds to half-siblings), and agrees with the theory (Eq (1)). The plot also depicts the expected distribution under the Poisson model, and demonstrates that the effect of interference can be substantial and is noticeable up to *T* ≲ 4. However, by *T* = 6 the Poisson process is already an excellent approximation to SA+intf.

Next we considered the effect of SS maps, this time assuming Poisson crossover placement. For concreteness, consider (*T* − 1)^th^-full cousins, which are separated by 2*T* meioses as above. Each IBD segment descends from one of the founder parents (a female or a male with equal probability), who transmits it via two meioses. The remaining 2*T* − 2 meioses can be either male or female with equal probability. The number of female transmissions is thus *n*_*f*_ = *n*_*f*,*i*_ + 2*n*_*f*,*a*_, where *n*_*f*,*i*_ is the number of female meioses that relate the full cousins ignoring the common ancestors, and is binomial with parameters (2*T* − 2, 1/2); and *n*_*f*,*a*_ indicates whether the common ancestor who transmitted the segment is female, and is Bernoulli with parameter 1/2. The number of male transmissions is *n*_*m*_ = 2*T* − *n*_*f*_. In fact, the same expressions hold for half-cousins, if the sex of the founder parent is random.

For each SNP *i*, denote by λ_*m*_(*i*) the male crossover rate (in Morgans per base-pair [bp]) between SNPs *i* and *i* + 1, and define λ_*f*_(*i*) similarly. We assume the rate is constant between SNPs (and zero before the first SNP). Given *n*_*f*_ and *n*_*m*_ female and male meioses, respectively, the total crossover rate between SNPs *i* and *i* + 1 for the two relatives is λ(*i*) = λ_*f*_(*i*)*n*_*f*_ + λ_*m*_(*i*)*n*_*m*_. Thus, placement of crossovers is still based on a Poisson process, but because the per bp rates in males and females differ by position, the rate is inhomogeneous along the genome. (Note that the male and female maps themselves are also inhomogeneous with respect to physical positions. We focus here on physical positions because the effects of the process ultimately occur at a physical position, and those physical positions are common to both the male and female maps).

To obtain the distribution of inter-crossover distances (again in physical bp) for a fixed number of male and female transmissions, we use a result by Yakovlev et al. [43] for the distribution of inter-event times in an inhomogeneous Poisson process. Denote λ(*x*) as the implied crossover rate at a physical coordinate *x* (as implied by the λ(*i*)’s above), and define $\Lambda \left(x\right)=\int_{0}^{x}\lambda \left(u\right)du$. Then we have

$$\begin{array}{c} \phi \left(x\right)=\frac{1}{\Lambda (L)}\int_{0}^{L-x}\lambda \left(y\right)\lambda \left(y+x\right)e^{-[\Lambda (y+x)-\Lambda (y)]}dy+\frac{\lambda \left(x\right)e^{-\Lambda (x)}}{\Lambda (L)}. \end{array}$$ (2)

Here *ϕ*(*x*) describes the density of all inter-crossover distances, not including the one censored by the chromosome end, with the number of male and female transmissions assumed given. To obtain the density without conditioning on male and female meiosis counts, we sum over all *n*_*f*_ = 0, …, 2*T*, each weighted by its probability. In relatives, not all inter-crossover blocks will become IBD segments, but rather only those whose line of descent is from the same common ancestor in both relatives. However, since IBD segments are a random subset of all blocks, the IBD segment length distribution is expected to be similar to that of the inter-crossover distances. This is confirmed in S15 Fig, where we plot the distribution of simulated IBD segment lengths under SS+Poiss for half-cousins separated by *T* = 1, 2, 4, 6 generations. As opposed to the observations from crossover interference, the distribution of segment lengths under SS maps is not substantially different from that obtained by Eq (2) with SA maps.

### Ped-sim comparison to IBDsim

The functionality in Ped-sim is available with some limitations in IBDsim [31], an R package that uses the *χ*^2^ interference model with fixed parameters. We used Ped-sim (under the SS+intf model) and IBDsim to simulate 10,000 full sibling pairs and 10,000 second cousin pairs (Methods). Ped-sim simulated the full siblings and second cousins in 8.1 and 8.7 seconds, respectively, while IBDsim required 371 and 608 seconds, respectively (corresponding to 46-fold and 70-fold speedups). Memory requirements are low for both methods, with Ped-sim and IBDsim using, respectively 0.62 Gb and 2.0 Gb to simulate the second cousins.

Neither of the above analyses produced genotype data (and IBDsim does not provide this functionality), but only generated IBD segments from replicate pedigrees. To produce genotype data, Ped-sim compute times are on the scale of dozens of minutes for several thousand samples. For example, simulating genotype data for 4,450 pairs of full siblings at 462,828 markers took 33.5 minutes for non-gzipped input and output (I/O) data and 59.2 minutes for gzipped I/O, and both runs used 0.13 Gb of RAM (Methods).

## Discussion

Modeling relatedness among individuals is more challenging than is typically appreciated due to the complexities of meiotic biology. Variable crossover rates between the sexes and the phenomenon of crossover interference both affect the quantity and size of IBD segments that individuals share. Our analyses demonstrate that use of sex-specific maps and inclusion of crossover interference provides the best fit to the standard deviation of IBD sharing rates in real human data from full siblings. Likewise, the 25th and 75th percentiles of IBD proportion from real first through second cousins are best fit by jointly modeling sex-specific maps and interference.

In modeling both the IBD sharing proportion between relatives and the lengths of their IBD segments, crossover interference has a much stronger influence than varying sex-specific versus sex averaged maps. However, sex-specific maps have a sizable impact on the number of IBD segments that both close (especially half-siblings) and somewhat distant (up to sixth cousins) relatives share. Therefore, both crossover interference and sex-specific maps play roles in the accurate representation of meiotic transmissions. Even so, the crossover model that is most discrepant with real IBD sharing fractions is the one that adopts sex-specific maps but Poisson inter-crossover distances. Thus, although closer to the true meiotic process in terms of the number of crossover events in men and women, the strong impact of interference in reducing the variance of IBD sharing is important to include when simulating relatives under a sex-specific map.

Given the effects on IBD sharing of the features we consider here, it is necessary to revisit the probability that a pair of relatives share any IBD segments with each other. A classic, influential treatment of this problem used an analytical approach based on Markov models and considered sex averaged maps while ignoring interference [18]. That study estimated that 10.1% of sixth cousins share IBD regions, which is close to the 9.9% we obtain using a more up-to-date sex averaged genetic map. Still, with both sex-specific maps and interference modeling, we find that 11.1% of simulated sixth cousins have non-zero IBD sharing. This factor rises to 13.1% when the sixth cousins are related primarily through females, and drops to 9.0% when they are related primarily through males.

A question that arises in light of these non-standard models is whether and in what contexts existing inference and/or simulation frameworks should incorporate the more realistic features. We examined the performance of degree of relatedness inference using KDEs trained with data simulated under the four crossover models. This analysis showed that the models trained under interference have slightly improved probability calibration for close relatives (S13 Fig). Perhaps more importantly, training with data from the traditional sex averaged map with Poisson localization has slightly decreased specificity overall and lower sensitivity for fifth and sixth degree relatives (S12 Fig).

A caveat to these and other results is that they leverage simulated, exact IBD segments, and the limitations of IBD detection in real data may make the signals we highlight impossible to reliably identify in practice. As described in Results, IBD detectors are affected by false positives and false negatives, and even without these concerns, real data include background IBD sharing that can confound downstream analyses. The approaches we employed of mean-shifting IBD sharing rates and, for the SAMAFS-validated data, limiting to pairs with rich evidence supporting their relationship type are infeasible in most studies. Further work will be needed to determine how practically useful the findings presented in this paper are. Still, some applications of non-standard crossover models have been developed, as the method CREST now uses sex-specific maps to infer whether real half-sibling and grandparent-grandchild pairs are maternally or paternally related using only their autosomal IBD segments [44].

Besides influencing relatedness, crossover models are a central feature of linkage analysis—an approach that computes the likelihood of trait association based on the co-segregation of alleles and traits within pedigrees. Several methods for linkage analysis already incorporate sex-specific maps [39, 45, 46], and use of these maps does impact linkage signals, both increasing and decreasing evidence of association depending the data used [47, 48]. By contrast, crossover interference is computationally intensive to model, and methods to perform such modeling have only been applied to very small datasets—both in numbers of meioses and markers [49, 50]. One study showed that accounting for interference can increase linkage analysis power [51], but this conclusion is based on simulated data with few markers. Analyses of the impact of interference on likelihood calculations using more recent high density SNP datasets may be worthwhile, and could be coupled with efforts to improve the speed of interference-based likelihood calculations.

In general, the comparisons most affected by the crossover properties considered here are between relatives separated by meiosis counts ≲ 12 (or *T* ≲ 6 in Fig 6, S15 Fig), while, for more distant relatives, it is reasonable to use traditional models. That is, while crossover models affect IBD sharing in even moderately distant relatives such as fifth or sixth cousins, population-based coalescent simulations and inference techniques are reasonable to perform with standard approximations.

Going forward, efforts to better understand the dynamics of crossovers, including observed “gamete effects” wherein crossover counts in a given gamete are correlated across chromosomes [52], and incorporating genetic variants that affect crossover rates [52] could yield models with even greater precision than those we focus on in this study. Nevertheless, the effects of crossover interference and sex-specific maps merit consideration in models of relatedness, as they alter IBD distributions in even moderately distant relatives.

## Methods

We analyzed data from a combination of simulated and real relatives, the latter from the SAMAFS and 20,240 full sibling pairs from Hemani et al. [35]. The IBD sharing statistic we focus on primarily is the proportion of their genome two relatives share IBD, calculated as a fraction of the diploid genome. For a given pair, this proportion is (*k*^(2)^ + *k*^(1)^/2), where *k*^(2)^ and *k*^(1)^ are the fraction of positions (in genetic map units) the pair shares IBD2 and IBD1, respectively. To perform degree of relatedness classification (S9 Fig), we used kinship coefficients calculated for a given pair as (*k*^(2)^/2+ *k*^(1)^/4)—i.e., 1/2 the IBD proportion—and mapped these coefficients to degrees according to the ranges KING uses [41].

### The Ped-sim algorithm

Ped-sim simulates relatives by tracking haplotypes—initially ignoring genetic data—as a sequence of segments that span a chromosome. Each segment consists of a numerical identifier denoting the founder haplotype it descends from and a segment end point. The start position is implicitly either the beginning of the chromosome or the site following the end of the previous segment. All founders have two haplotypes with only one chromosome-length segment, each with a unique identifier.

To begin, Ped-sim reads a file that defines the pedigree structure(s) it is to simulate and, for each such structure, generates haplotype segments for the founders in the first generation. For subsequent generations, it generates haplotypes for any founders in that generation and forms haplotypes for non-founders from the parents’ haplotypes under a meiosis model. This model works on the two haplotypes belonging to a given parent by first randomly selecting which of these begins the offspring haplotype, each having 1/2 probability of being selected. Next, Ped-sim samples the location of the crossover events, either using a model of crossover interference or a Poisson model. It then produces the offspring haplotype by copying the segments that comprise the parent’s initial haplotype up to the position of the first crossover, and introduces a break point in the copied segment at that crossover position. Following this, it switches to copying segments from the parent’s other haplotype, and it continues to alternate copied-from haplotypes at each crossover in this manner until the end of the chromosome.

Details of the Housworth-Stahl crossover interference model are below, and we discuss parameter choices in the next subsection.

Under the Poisson crossover model, the distance from the start of the chromosome to the first crossover, and from one crossover to the next are each exponentially distributed with rate equal to 1 crossover/Morgan. This rate arises naturally from the definition of a Morgan as the distance within which an average of one crossover occurs per generation. The model sequentially samples crossovers and terminates after sampling a crossover beyond the end of the chromosome.

Both models produce crossover positions in genetic units (i.e., Morgans), and Ped-sim determines their physical location using a genetic map, storing the segment end points as physical bp positions. When using sex-specific maps, it locates the physical positions using the map corresponding to the sex of the parent. If a crossover falls between two defined map positions, Ped-sim uses linear interpolation to determine the physical location.

By default, Ped-sim randomly assigns the sexes of parents, and can generate any number of pedigrees with a given structure (with parent sexes assigned independently in each). Ped-sim can also generate data in which all reproducing non-founders have the same sex (male or female), leading to descendants that are related to each other through nearly all male or all female relatives. In such cases, the common ancestors of those descendants will generally be a couple (S8B Fig), male and female, although it is possible to simulate only a single common ancestor or for individuals to be related through more than one lineage (e.g., double first cousins).

When given genetic data in the form of input haplotypes, Ped-sim randomly assigns the data from one input sample to each founder. It then copies alleles from these assigned founder haplotypes to descendants using the segment numerical identifiers and end points. The algorithm can also introduce genotyping errors and missing data using user-specified rates, with a uniform probability of these events at all positions. The genotyping error model only applies to biallelic sites, and Ped-sim does not introduce errors at multi-allelic variants. When assigning an erroneous genotype at a truly heterozygous site, Ped-sim changes the marker to be either of the two homozygous genotype classes with equal probability. For errors at truly homozygous markers, Ped-sim converts the site to be heterozygous under the default settings, but it also has a probability of changing the site to the opposite homozygous genotype (with a default rate of 0).

Another way to run Ped-sim is without haplotype data—instead using only IBD segments detected using the internally tracked haplotype segments. This is the way we ran Ped-sim for the analyses we describe here, using version 1.0.1 of the tool unless otherwise specified. The IBD segments Ped-sim generates consist of physical start and end positions in both physical (bp) and genetic units (cM). To be able to analyze statistics of IBD sharing in terms of genetic distances, Ped-sim reports the sex averaged genetic start and end positions of each segment (the midpoint of the sex-specific coordinates), even when using sex-specific maps.

Ped-sim is available from https://github.com/williamslab/ped-sim, with several example pedigree definition (def) files and the interference parameters we used [15] included in the repository. The documentation in the repository includes links and Bash code for downloading and generating a file with the sex-specific map we used [16].

### Genetic maps and crossover interference parameters

We used genetic maps produced using crossovers from over 100,000 meioses [16]. These maps include those for both males and females and a sex averaged map that all span the same physical range. All simulations include the 22 autosomes but no sex chromosomes.

To simulate using the Housworth-Stahl crossover interference model, we leveraged female and male interference parameters *ν*_*i*_ and *p*_*i*_ for *i* ∈ {*f*, *m*}, respectively, that were inferred from over 18,000 meioses [15]. We calculated the sex averaged parameters *ν*_*a*_ and *p*_*a*_ as follows. The *p* parameter gives the fraction of events that escape interference, and we set *p*_*a*_ = (*p*_*f*_ + *p*_*m*_)/2. In this model, distances between chiasmata subject to interference are gamma distributed with shape and rate parameter values *ν* and 2(1 − *p*)*ν*, respectively [13, 14]. A simple average of the male and female *ν* parameters does not produce a distribution with summary statistics at the midpoint between the two sexes. All values of *ν* lead to distributions with the same expected value of $\frac{1}{2(1-p)}$ because the expectation of a gamma distribution is the shape divided by the rate parameter. We therefore calculated *ν*_*a*_ such that the variance of the sex averaged distribution is the mean of the variances of the male and female models, while assuming all *p* parameters are the same between the models (since we separately estimate *p*_*a*_). This gives $\nu_{a}={\left(\frac{1}{2\nu_{f}}+\frac{1}{2\nu_{m}}\right)}^{-1}$. Note that the mean distance of 1/2 Morgans between events (accounting for both the regulated crossovers and those that escape interference) is half the distance expected per chromatid. This is because the model is for events in a tetrad (all four products of meiosis). To obtain the crossovers falling on the gamete being generated, the model randomly selects events with probability 1/2 for inclusion.

### IBD sharing fractions between full siblings in simulated nuclear families

To evaluate the reliability of full sibling IBD sharing fractions estimated using family-based phasing of data from nuclear families, we used Ped-sim 1.0.2 to simulate data for 500 nuclear families with three children and 500 nuclear families with five children. These simulations resulted in 1,500 full sibling pairs from three child families and 5,000 pairs from five child families, and we used the SS+intf model with default error and missing data rates (10^−3^ per site for each). The founder haplotypes input to Ped-sim are a subset of data from a multiple sclerosis case-control study [53] consisting of 8,955 samples typed at 462,828 markers. These data were previously used to evaluate a multi-way relatedness inference method; filters and phasing procedures used to generate it are in the corresponding paper [6]. We used the same approach to infer IBD fractions in these simulated siblings as in the SAMAFS data (outlined in the next subsection), and for each pair we calculated the difference between the predicted and true IBD fractions (S1 Fig).

### IBD detection in the SAMAFS

We used two different methods for inferring IBD fractions in the SAMAFS data: one applied to nuclear families and which we used to analyze IBD sharing between full siblings, and the other for analyzing IBD rates in first cousins, first cousins once removed, and second cousins. Quality control filtering of the SAMAFS data is the same as that described previously [54, 55]. In brief, we used biallelic SNPs typed on the Illumina Human660W, Human1M, Human1M-Duo, or both the HumanHap500 and the HumanExon510S arrays, and required the SNP probe sequences to map to a single location in the human GRCh37 build. Next, we excluded individuals and SNPs with excessive missing data (>10% and >2%, respectively) and removed duplicate SNPs. Additional SNP filters utilized information from auxiliary resources including dbSNP and the reported “accessible genome” from the 1000 Genomes Project, among others [54]. This yielded data for 2,485 samples typed at 521,184 SNPs. We further omitted 1,514 first cousin, first cousin once removed, and second cousin relative pairs that had evidence of being related through more than one lineage [55].

Family-based phasing implicitly infers IBD regions, and in the presence of data for a complete nuclear family, this inference is very accurate, as explained in Results and demonstrated using simulated data (S1 Fig). For this analysis, we utilized HAPI [38] version 1.89.2, a method that performs efficient minimum recombinant phasing for nuclear families. This form of phasing is the same as that of the Lander-Green algorithm [56] when the probability of crossover between informative markers is identical at each position. To ensure reliable results, we performed this inference on 116 nuclear families for which data from both parents and three or more children were available, and we excluded one member of likely monozygotic twin pairs that had IBD2 rates >0.95 (three pairs). We also ran with the --no_err_max 1 option, which filters sites where one or more children inherit a recombination relative to the previous site and where the next informative site reverts to the original transmitted haplotypes.

To infer IBD regions, we parsed the inheritance vector output from HAPI to locate IBD1 and IBD2 segments, assigning genetic positions to the start and end of each such region using the same sex averaged map we used for the simulated data [16]. (The genetic map is undefined for 102 SNPs and we omitted these positions from analysis.) The exact boundaries of crossover positions are uncertain in real data due to the fact that not all sites are genotyped and positions that are homozygous in a parent are uninformative. We therefore estimated the start and end positions as the midpoint in genetic units between two informative sites that descend from distinct parental haplotypes and therefore bound the region in which a crossover broke an IBD segment. To ensure the IBD intervals are real and not due to genotyping error, we also merged short regions (including non-IBD intervals) comprised of < 10 informative SNPs with the adjacent segments so that they cover the interval. We assign these to have the same IBD type as the preceding segment (typically the two flanking segments are of the same type). On average each pair had 6.75 merged regions across all autosomes (S16 Fig), and we removed pairs that had more than 100 merged regions. This filter removed a total of eight pairs from three families. Given this enrichment, we removed all siblings in these families from the analysis, leading to the further exclusion of seven full sibling pairs. Finally, we removed two outlier full sibling pairs that had low IBD proportions of 0.356 and 0.368. This yielded 1,128 pairs of full siblings for analysis.

For non-sibling relatives, we leveraged IBD segments previously inferred [55] using Refined IBD [37] version 4.1. In total, the SAMAFS relatives consist of 5,384 pairs of first cousins, 6,342 first cousins once removed, and 2,584 second cousins. Here as well we converted physical positions of the IBD segments to sex averaged genetic positions using the same sex averaged map as in other analyses [16].

While the SAMAFS relationships are reliable [55], the statistics we consider are sensitive and can be meaningfully affected by small numbers of mislabeled pairs. We therefore sought to identify a validated set of relative pairs whose relationships are nearly certain to be correct. For validation, we required pairs to be descended from a pair of full siblings that are genotyped and inferred as first degree relatives by Refined IBD. We further required data for all parent-child relatives that fall between those full sibling ancestors and their descendants, and ensured that Refined IBD inferred the parent-child pairs as first degree relatives (S8A Fig). Subsetting the data in this way produces a validated set of 3,722 first cousins, 2,869 first cousins once removed, and 906 second cousins. Only one pair of the full sibling ancestors were not inferred as first degree relatives by Refined IBD, while Refined IBD inferred all the relevant parent-child pairs as first degree relatives.

We noted that the mean IBD rates for the real relatives are slightly elevated, potentially due to background relatedness or false positive IBD segments. For first cousins, the mean amount of IBD shared exceeds the theoretical expectation by 11.3 cM, and 19.5 cM in the validated set (0.17% and 0.29% above the expectation, respectively). For first cousins once removed, the observed means are greater than supported by theory by 0.17 cM (0.0026%), and 15.9 cM (0.24%) for the validated set. Finally, the excess for second cousins is 17.6 cM (0.26%), and 6.8 cM (0.10%) for the validated set. We subtracted off these mean excesses for each of these (non-sibling) relationship types in Fig 1, S2 and S3 Figs and associated analyses; the unmodified statistics are in S1 Dataset. We did not mean-shift the kinship coefficients used to map the SAMAFS samples to degrees of relatedness (S9 Fig).

### Number of standard errors separating real and simulated data

To compare IBD sharing statistics from the real and simulated data (including the standard deviations, 25th and 75th percentiles [Fig 1], and the rates of inferring pairs to their true degree of relatedness [S9 Fig]), we quote distances in terms of number of standard errors that separate the values. We calculated this distance as

$$\begin{array}{c} D=\frac{\theta_{\text{model}}-\theta_{\text{real}}}{\sqrt{{\text{SE}}_{\text{model}}^{2}+{\text{SE}}_{\text{real}}^{2}}}, \end{array}$$

where *θ*_model_ and *θ*_real_ are point estimates of the statistic being compared in the simulated and real data, respectively, and *SE*_model_ and *SE*_real_ are the corresponding simulated and real standard errors of *θ*, respectively. We obtain these standard errors by bootstrapping with 1,000 samples.

### IBD detection in SAMAFS half-siblings

To detect IBD segments shared between SAMAFS half-siblings (S11B Fig), we identified pairs listed as half-siblings in the SAMAFS pedigrees and contained in the 2,485 samples used for other analyses. Next, we retained pairs Refined IBD inferred as second degree relatives [55]. We then ran IBIS [57] version 1.02 to detect IBD segments using genetic positions from the SA map and default parameter settings (minimum segment length of 7 cM, minimum number of markers per segment of 500, and an error threshold of 0.004).

### Using kernel density estimation to infer degrees of relatedness

We generated four degree of relatedness classifiers based on KDEs, each trained using simulated data from one of the four Ped-sim models (here using Ped-sim version 1.0.2). For each classifier, the training data consist of 4,000 pairs of relatives that share IBD with each other of each degree from first to seventh, i.e., from parent-child to third cousin pairs. We included two relative types for each degree—one in which the pair shares two common ancestors (a couple), and the other in which they share one common ancestor. (We consider full sibling and parent-child pairs for first degree relatives.) The KDEs use the IBD sharing fraction and number of segments a pair shares as features, and we rescaled each feature to have range between 0 and 1 by dividing by the maximum value in the training data. We used five-fold cross validation to decide the optimal bandwidth and kernel function for each degree.

To test the classifiers, we used an independent set of 4,000 first through sixth degree pairs simulated under the SS+intf model. Note that we do not report accuracy results for seventh degree relatives; we included seventh degree relationships in the classifiers to act as an “unrelated” class that provides bounds on sixth degree relatedness classification. The estimated density functions enable calculation of the posterior probability that each pair of relatives belongs to a given degree (where we use a uniform prior), and we classified relatives to their maximum posterior probability degree. We also generated calibration curves to evaluate the reliability of these predicted probabilities for each classifier.

### Estimating time since admixture corrected for finite chromosomes

We simulated admixed individuals using the pedigree structure depicted in S14 Fig, wherein half the first generation ancestors descend from one source population and half from another. We identified local ancestry segments by inspecting the IBD segments that the admixed individuals share with these first generation ancestors and merging adjacent segments that descend from the same population. To estimate the time since admixture based on the resulting local ancestry segment lengths, we assumed the segments derive from a Poisson process—and thus segment lengths follow an exponential distribution—and used the following maximum likelihood approach.

Suppose a two-way admixture event occurred *T* generations ago, with all couples in that generation including one member of each population (S14 Fig). Note that crossovers only introduce ancestry switches when a parent is heterozygous for ancestry, which will be the case for half (on average) of such crossovers for the more recent *T* − 2 meioses since, on average, these ancestors will inherit half their genome from each population [58]. At the same time, for individuals in the second generation, all positions are heterozygous for ancestry, so all crossovers produce ancestry switches. Thus, the Poisson rate at which ancestry switches occur is (*T* − 2)/2 + 1 = *T*/2 per Morgan, and the likelihood of a segment of Morgan length *x* is hence (*T*/2)*e*^−*Tx*/2^ Eq (1) in [58]. However, segments bounded by the end of the chromosomes are “censored,” and we only know they are longer than *x*, and thus they have likelihood *e*^−*Tx*/2^. Assuming the segments are independent, the likelihood of all segments is ${\left(T/2\right)}^{n-m}e^{-\left(T/2\right)\sum_{i=1}^{n}x_{i}}$, where there are *n* segments of lengths *x*_1_, …, *x*_*n*_, *m* of which are censored by chromosome ends. Equating the derivative of the log-likelihood to zero, we obtain the maximum likelihood estimate $\hat{T}=2\left(n-m\right)/\sum_{i=1}^{n}x_{i}$.

### Deriving the distribution of IBD segment lengths under the Housworth-Stahl interference model

#### Background

We assume a sex-averaged genetic map and that the chromosome is infinitely long (we will relax this assumption later). Under the Housworth-Stahl two-pathway model [14], a proportion *p* of crossovers escape regulation (referred to as “free” crossovers below), and are thus distributed along the chromosome as a Poisson process with rate *p* per Morgan. Regulated crossovers are generated independently of the unregulated crossovers by first drawing the positions of chiasmata as a stationary renewal process [42] along the chromosome, with gamma distributed inter-chiasma distances (in Morgans) with shape *ν* and rate 2*ν*(1 − *p*). Then, each chiasma becomes a crossover on the modeled gamete with probability 1/2, since it affects only one of the two sister chromatids. The latter process is called thinning and assumes no chromatid interference. The average inter-crossover distance is 1 Morgan, as per the definition of the Morgan unit.

In the regulated process, if *k* − 1 chiasmata are skipped between crossovers, the distance to the next crossover is distributed as gamma with shape *kν* and rate 2(1 − *p*)*ν*. After thinning, the distance between regulated crossovers is distributed as

$$\begin{array}{c} f_{\text{reg}}\left(x\right)=\sum_{k=1}^{\infty }{\left(\frac{1}{2}\right)}^{k}\frac{x^{k\nu -1}e^{-2(1-p)\nu x}{\left[2\left(1-p\right)\nu \right]}^{k\nu }}{\Gamma (k\nu )}. \end{array}$$ (3)

The free process has inter-crossover distances distributed as

$$\begin{array}{c} f_{\text{free}}\left(x\right)=pe^{-px}. \end{array}$$ (4)

Below, we study the distance between crossovers across 2*T* meioses. As explained in Results, the IBD segment length distribution is expected to be similar to that of the inter-crossover distance distribution, as IBD segments are a random subset of the inter-crossover regions. To proceed, we will first compute the properties of the distance from a fixed point to the nearest downstream crossover, and then use these results to derive the distribution of inter-crossover distances across multiple meioses.

#### The distance from a fixed point to a crossover in one meiosis

The process of placing chiasmata is assumed to be a stationary renewal process [14]. In one meiosis, the distance between a randomly selected site and the next crossover to the right (or left) due to the regulated process is distributed as [42]

$$\begin{array}{c} g_{\text{reg}}\left(x\right)=\frac{1}{\mu_{\text{reg}}}[1-F_{\text{reg}}\left(x\right)], \end{array}$$ (5)

where *F*_reg_(*x*) is the CDF of *f*_reg_(*x*), i.e., $F_{\text{reg}}\left(x\right)=\int_{0}^{x}f_{\text{reg}}\left(y\right)dy$, and *μ*_reg_ is the mean distance between regulated crossovers (i.e., $\mu_{\text{reg}}=\int_{0}^{\infty }xf_{\text{reg}}\left(x\right)dx$). As $\mu_{\text{reg}}=1/\left(1-p\right)$, we have

$$\begin{array}{c} g_{\text{reg}}\left(x\right)=\left(1-p\right)[1-F_{\text{reg}}\left(x\right)]. \end{array}$$ (6)

This can be written explicitly as

$$\begin{array}{c} g_{\text{reg}}\left(x\right)=\left(1-p\right)\int_{x}^{\infty }f_{\text{reg}}\left(y\right)dy\\ =\left(1-p\right)\int_{x}^{\infty }\sum_{k=1}^{\infty }\frac{2^{-k}y^{k\nu -1}e^{-2(1-p)\nu y}{\left[2\left(1-p\right)\nu \right]}^{k\nu }}{\Gamma (k\nu )}dy. \end{array}$$ (7)

Changing variables, *z* = 2(1 − *p*)*νy*, we obtain

$$\begin{array}{cc} g_{\text{reg}}\left(x\right) & =\left(1-p\right)\sum_{k=1}^{\infty }\frac{2^{-k}}{\Gamma (k\nu )}\int_{2(1-p)\nu x}^{\infty }z^{k\nu -1}e^{-z}dz\\ & =\left(1-p\right)\sum_{k=1}^{\infty }2^{-k}\frac{\Gamma [k\nu ,2(1-p)\nu x]}{\Gamma (k\nu )}, \end{array}$$ (8)

where Γ[*a*, *x*] is the upper incomplete gamma function. We denote the CDF of *g*_reg_(*x*) as *G*_reg_(*x*).

For the free process, as it is memory-less,

$$\begin{array}{c} g_{\text{free}}\left(x\right)=pe^{-px}, \end{array}$$ (9)

and the CDF of *g*_free_(*x*) is

$$\begin{array}{c} G_{\text{free}}\left(x\right)=1-e^{-px}. \end{array}$$ (10)

#### The distance from a fixed point to a crossover across multiple meioses

The previous subsection described the distance to the next crossover of a given type (regulated/free) for a single meiosis. Given a focal site, the distance to the next crossover across 2*T* meioses and both processes is the minimum of the distance to 2*T* regulated processes and 2*T* free processes. We model all processes as independent, which is clearly true for meioses in different individuals, and holds for the regulated and free processes under the Housworth-Stahl model. Thus, the distance to the next crossover across 2*T* meioses is distributed as

$$\begin{array}{cc} h(x) & =2T{\left[1-G_{\text{reg}}\left(x\right)\right]}^{2T-1}{\left[1-G_{\text{free}}\left(x\right)\right]}^{2T}g_{\text{reg}}\left(x\right)\\ & +2T{\left[1-G_{\text{reg}}\left(x\right)\right]}^{2T}{\left[1-G_{\text{free}}\left(x\right)\right]}^{2T-1}g_{\text{free}}\left(x\right). \end{array}$$ (11)

In the first term, all free crossovers and all but one of the regulated crossovers have distance larger than *x*, and one of the regulated crossovers has distance *x*. There are 2*T* possibilities to choose which regulated crossover has the minimal distance, and hence the initial factor of 2*T*. The second term is similar, with the minimal distance now coming from one of the free crossovers. Eq (11) can be simplified based on *g*_free_(*x*) and *G*_free_(*x*) from Eqs (9) and (10) as

$$\begin{array}{c} h\left(x\right)=2Te^{-2pTx}{\left[{\tilde{G}}_{\text{reg}}\left(x\right)\right]}^{2T-1}(g_{\text{reg}}\left(x\right)+p{\tilde{G}}_{\text{reg}}\left(x\right)), \end{array}$$ (12)

where ${\tilde{G}}_{\text{reg}}\left(x\right)=1-G_{\text{reg}}\left(x\right)=\int_{x}^{\infty }g_{\text{reg}}\left(y\right)dy$.

#### The length of a randomly chosen inter-crossover distance

Denote by *ϕ*(*x*) the density of a randomly chosen inter-crossover distance. Cox and Smith [59] proved a result on superposition of renewal processes that applies to our case. According to their Eq. (31), if the density of the distance to the next event (crossover) across all processes (meioses) is *h*(*x*), then the density of the length of a randomly chosen inter-crossover interval is given by

$$\begin{array}{c} \phi \left(x\right)=-\frac{\partial h(x)}{\partial x}\cdot \langle x\rangle , \end{array}$$ (13)

where 〈*x*〉 is the mean inter-crossover length (across all meioses). In our case, 〈*x*〉 = 1/(2*T*), and thus

$$\begin{array}{c} \phi \left(x\right)=-\frac{1}{2T}\frac{\partial h(x)}{\partial x}. \end{array}$$ (14)

Substituting Eq (12), and using the facts that $-\frac{\partial }{\partial x}{\tilde{G}}_{\text{reg}}\left(x\right)=g_{\text{reg}}\left(x\right)$ and $-\frac{\partial }{\partial x}g_{\text{reg}}\left(x\right)=\left(1-p\right)f_{\text{reg}}\left(x\right)$,

$$\begin{array}{cc} \phi (x) & =-\frac{1}{2T}\frac{\partial }{\partial x}\{2Te^{-2pTx}{\left[{\tilde{G}}_{\text{reg}}\left(x\right)\right]}^{2T-1}(g_{\text{reg}}\left(x\right)+p{\tilde{G}}_{\text{reg}}\left(x\right))\}\\ & =-\frac{\partial }{\partial x}\{e^{-2pTx}{\left[{\tilde{G}}_{\text{reg}}\left(x\right)\right]}^{2T-1}g_{\text{reg}}\left(x\right)\}-p\frac{\partial }{\partial x}\{e^{-2pTx}{\left[{\tilde{G}}_{\text{reg}}\left(x\right)\right]}^{2T}\}\\ & =e^{-2pTx}{\left[{\tilde{G}}_{\text{reg}}\left(x\right)\right]}^{2T-1}\left[2pTg_{\text{reg}}\left(x\right)+\frac{\left(2T-1\right)g_{\text{reg}}^{2}\left(x\right)}{{\tilde{G}}_{\text{reg}}\left(x\right)}+\left(1-p\right)f_{\text{reg}}\left(x\right)+2p^{2}T{\tilde{G}}_{\text{reg}}\left(x\right)+2pTg_{reg}\left(x\right)\right]\\ & =e^{-2pTx}{\left[{\tilde{G}}_{\text{reg}}\left(x\right)\right]}^{2T-1}\left[4pTg_{\text{reg}}\left(x\right)+\frac{\left(2T-1\right)g_{\text{reg}}^{2}\left(x\right)}{{\tilde{G}}_{\text{reg}}\left(x\right)}+\left(1-p\right)f_{\text{reg}}\left(x\right)+2p^{2}T{\tilde{G}}_{\text{reg}}\left(x\right)\right]. \end{array}$$ (15)

Eq (15) is our main result, and is summarized as Eq (1) in Results. We note that as opposed to the Poisson model, inter-crossover distances under interference in a single meiosis are not independent, which is a general property of a superposition of renewal processes. To evaluate the various terms in Eq (15) in our simulations, we truncated all sums at *k* = 50 and calculated all integrals using Matlab’s integral function.

#### Finite chromosomes

The results above apply only to infinite-length chromosomes. To determine the distribution of segment lengths for finite chromosomes, we use a result derived by Gravel [58] in the context of local ancestry segments. Gravel showed that if a process along the chromosome partitions it into segments with a stationary length density *ϕ*(*x*), the density of segment lengths in a finite chromosome, *ϕ*_*L*_(*x*), is given by

$$\begin{array}{c} \phi_{L}\left(x\right)=\frac{2\int_{x}^{\infty }\phi \left(y\right)dy+\left(L-x\right)\phi \left(x\right)+\delta \left(L-x\right)\int_{L}^{\infty }\left(y-L\right)\phi \left(y\right)dy}{L+\int_{0}^{\infty }y\phi \left(y\right)dy}, \end{array}$$ (16)

where *δ*(*L* − *x*) is the Dirac delta function, representing the probability that *x* spans the entire chromosome.

The theoretical distribution for the Poisson model, for an infinitely long chromosome, is *ϕ*(*x*) = 2*Te*^−2*Tx*^. Applying the finite chromosome correction of Eq (16), we obtain

$$\begin{array}{c} \phi_{L}\left(x\right)=\frac{2Te^{-2Tx}\left[2+2T\left(L-x\right)\right]+e^{-2TL}\delta \left(L-x\right)}{2TL+1}. \end{array}$$ (17)

### Runtime analyses

To collect runtime statistics, we ran Ped-sim 1.0.1 and IBDsim 0.9-8 on a machine with four Xeon E5 4620 2.20GHz CPUs and 256 GB of RAM. We report wall clock time averaged from three runs of each program to produce IBD segments from 10,000 full siblings and 10,000 second cousins. To simulate the 10,000 full siblings with IBDsim, we used the following R code:

library(IBDsim)

quad <- nuclearPed(2)

res <- IBDsim(quad, sims = 10000,

        query = list(’atleast1’ = 3:4))

To simulate the siblings in Ped-sim, we used the following def file:

def full-sibs 10000 2

2 1

IBDsim defaults to assigning all non-founders as male and their spouses as female. We simulated 5,000 second cousin pedigrees with the default non-founder sex assignment, and the other 5,000 with female non-founders using the following R code:

library(IBDsim)

second_cousin_nonfound_male <- cousinPed(2)

res_male <- IBDsim(second_cousin_nonfound_male, sims = 5000,

            query = list(’atleast1’ = 11:12))

second_cousin_nonfound_female <-

  swapSex(second_cousin_nonfound_male, c(3,4,7,8))

res_female <- IBDsim(second_cousin_nonfound_female, sims = 5000,

            query = list(’atleast1’ = 11:12))

For Ped-sim, we simulated the same second cousin pedigree structures with the def file:

def second-cous-male 5000 4 M

4 1

def second-cous-female 5000 4 F

4 1

To benchmark Ped-sim’s time to produce genetic data for 4,450 full sibling pairs, we ran version 1.0.2 of the tool under the SS+intf model using input haplotypes from the same 8,955 multiple sclerosis case-control samples described above [53] (see “IBD sharing fractions between full siblings in simulated nuclear families”), and otherwise used default Ped-sim options.

### Ethics statement

This study makes use of deidentified individuals from the SAMAFS and received exemption (#4) from IRB review from the Cornell University IRB (protocol 1408004874).

## Supporting information

S1 Fig Differences in true and predicted IBD sharing fractions of full siblings from simulated nuclear families. IBD sharing fractions are from the full sibling pairs of 500 simulated nuclear families with three children (left) and 500 with five children (right). We phased these families and extracted IBD sharing estimates as described in Methods.
(TIF)

S2 Fig IBD sharing fraction 25th and 75th percentiles in full siblings and standard deviations in first through second cousins from real and simulated data. Points are from the SAMAFS, SAMAFS-validated subset (except full siblings), Hemani20k set (only full siblings), and the simulation models. The latter are labeled using abbreviations given in the main text. Bars indicate 95% confidence interval (±1.96 standard errors) as calculated from 1,000 bootstrap samples. SD indicates standard deviation.
(TIF)

S3 Fig Mean, minimum, median, and maximum IBD sharing fractions in real and simulated data for full siblings through second cousins. Points are from the SAMAFS, SAMAFS-validated subset (except full siblings), Hemani20k set (only full siblings), and the simulation models. The latter are labeled using abbreviations given in the main text. The SAMAFS and SAMAFS-validated values are mean-shifted to match expectations for the first cousins, first cousins once removed, and second cousins, but are unaltered for the full sibling and the full sibling IBD2 quantities. Bars indicate 95% confidence interval (±1.96 standard errors) as calculated from 1,000 bootstrap samples.
(TIF)

S4 Fig First cousins simulated using sex-specific maps have visually similar distributions of IBD sharing fractions relative to those simulated under a sex averaged map. Sex-specific and sex averaged distributions heavily overlap both when using an interference (left) and a Poisson (right) model for inter-crossover distances.
(TIF)

S5 Fig Distributions of IBD sharing fractions for simulated full siblings and the real SAMAFS and Hemani20k full siblings. Each simulation includes 10,000 full sibling pairs, the SAMAFS data include 1,128 pairs (Methods), and the Hemani20k data total 20,240 pairs.
(TIF)

S6 Fig Overlay of SAMAFS full sibling IBD sharing distribution with those of simulated full siblings from each crossover model. Plots are histograms of the 1,128 SAMAFS pairs and 10,000 simulated pairs generated under each of the crossover models, as indicated.
(TIF)

S7 Fig Overlay of Hemani20k full sibling IBD sharing distribution with those of simulated full siblings from each crossover model. Plots are histograms of the 20,240 Hemani20k pairs and 10,000 simulated pairs generated under each of the crossover models, as indicated.
(TIF)

S8 Fig Example pedigree structures for SAMAFS-validated relatives and for relatives descended from female-only non-founders. (A) SAMAFS-validated pairs are required to be descended from a genotyped (black) full sibling pair and to have genotyped parent-child relatives that directly connect them to the full siblings. We further require that both the ancestral full sibling pair and all parent-child pairs be inferred as first degree relatives by Refined IBD. (B) Plot of female-lineage fourth cousins.
(TIF)

S9 Fig Rates of inferring real and simulated relatives to their true degree of relatedness. Degrees are inferred from kinship coefficients, with the latter calculated using inferred (for SAMAFS and SAMAFS-validated) or true (for the simulations) IBD segments (see Methods). Bars indicate 95% confidence interval (±1.96 standard errors) based on 1,000 bootstrap samples over relative pairs.
(TIF)

S10 Fig Detailed numbers of IBD segments that simulated third through sixth cousins share under various modeling scenarios. Percentages above each bar indicate the fraction of simulated relatives (of 10,000 for each scenario) that have at least one segment shared. Within stacked colored bars, numbers are the percentage of relatives that share the indicated number of IBD segments. Error bars above a given stacked bar is the 95% confidence interval (±1.96 standard errors) of the percentage of relatives that share the indicated number of segments based on 1,000 bootstrap samples.
(TIF)

S11 Fig Number of IBD segments maternal and paternal half-siblings share. (A) 10,000 simulated pairs for both types of half-siblings under the SS+intf model. (B) Number of IBD segments shared between maternal and paternal half-siblings within SAMAFS.
(TIF)

S12 Fig Classification rates for inferring degrees of relatedness using KDEs trained under the four different crossover models. The sensitivity (left) and specificity (right) of the classifiers, with the crossover model used to simulate the training data for each KDE indicated by line color. Rates are from 4,000 pairs of relatives in each degree, each simulated under the SS+intf model.
(TIF)

S13 Fig Calibration curves of the probabilities of classifying relatives to a given degree of relatedness using KDEs trained under the four different crossover models. We binned the predicted probabilities into bins of size 0.2. In each plot, the x-axis shows the per-bin mean predicted probability and the y-axis indicates the proportion of pairs that are of the given degree in the corresponding bin. The crossover model used to simulate the training data for each KDE is indicated by line color.
(TIF)

S14 Fig Pedigree structure used to simulate admixed samples. The number of generations since admixture, *T*, varies, and the number of unadmixed ancestors in the first generation is 2^*T*^/2. Plot shows *T* = 3 (ignoring ellipses) with paternal ancestors. The simulated ancestors are randomly either maternal or paternal. IBD segments between samples with filled shapes define local ancestry regions.
(TIF)

S15 Fig The effect of sex-specific maps on IBD segment lengths. We used Ped-sim to simulate half-cousins with a common ancestor *T* = 1, 2, 4, 6 generations ago (panels A-D, respectively) under the SS+Poiss model, extracting IBD segment lengths in bp for chromosome 1. Each panel shows the simulated distribution of IBD segment lengths (over 10^5^ pairs for *T* = 1, 2 and 10^6^ pairs otherwise; purple circles), the theory from Eq (2) (blue lines), and the expectation based on a sex-averaged map (red dashed lines). To evaluate Eq (2) we replaced the integrals with sums over discrete coordinates, evenly separated by 10^4^ bp.
(TIF)

S16 Fig Change in cM length shared and number of regions merged in the SAMAFS full siblings. Predicted IBD segments consisting of < 10 informative SNPs are potentially false, and we merged these with the previous segment (Methods). On average, this resulted in a total of 6.75 merged regions per pair across all autosomes, and an average increase of 0.0495 cM shared. No pair gained or lost more than 4.3 cM of IBD regions.
(TIF)

S1 Dataset Summary statistics and pairwise IBD proportions used to produce Figs 1, 3, S2, S3, S5, S6, S7, S9, S10, S11, S16 Figs, and results given in the text. (XLSX)

## References

[1] Weir BS, Anderson AD, Hepler AB. Genetic relatedness analysis: modern data and new challenges. Nature Reviews Genetics. 2006;7(10):771–780. 10.1038/nrg1960 16983373

[2] Bycroft C, Freeman C, Petkova D, Band G, Elliott LT, Sharp K, et al The UK Biobank resource with deep phenotyping and genomic data. Nature. 2018;562(7726):203–209. 10.1038/s41586-018-0579-z 30305743 PMC6786975

[3] Staples J, Maxwell EK, Gosalia N, Gonzaga-Jauregui C, Snyder C, Hawes A, et al Profiling and Leveraging Relatedness in a Precision Medicine Cohort of 92,455 Exomes. The American Journal of Human Genetics. 2018;102(5):874–889. 10.1016/j.ajhg.2018.03.012 29727688 PMC5986700

[4] Staples J, Witherspoon DJ, Jorde LB, Nickerson DA, Below JE, Huff CD, et al PADRE: Pedigree-Aware Distant-Relationship Estimation. The American Journal of Human Genetics. 2016;99(1):154–162. 10.1016/j.ajhg.2016.05.020 27374771 PMC5005450

[5] Ko A, Nielsen R. Composite likelihood method for inferring local pedigrees. PLOS Genetics. 2017;13(8):e1006963 10.1371/journal.pgen.1006963 28827797 PMC5578687

[6] Ramstetter MD, Shenoy SA, Dyer TD, Lehman DM, Curran JE, Duggirala R, et al Inferring Identical-by-Descent Sharing of Sample Ancestors Promotes High-Resolution Relative Detection. The American Journal of Human Genetics. 2018;103(1):30–44. 10.1016/j.ajhg.2018.05.008 29937093 PMC6035284

[7] Staples J, Qiao D, Cho MH, Silverman EK, Nickerson DA, Below JE. PRIMUS: Rapid Reconstruction of Pedigrees from Genome-wide Estimates of Identity by Descent. The American Journal of Human Genetics. 2014;95(5):553–564. 10.1016/j.ajhg.2014.10.005 25439724 PMC4225580

[8] Epstein MP, Duren WL, Boehnke M. Improved Inference of Relationship for Pairs of Individuals. The American Journal of Human Genetics. 2000;67(5):1219–1231. 10.1016/S0002-9297(07)62952-8 11032786 PMC1288564

[9] Hill W, Weir B. Variation in actual relationship as a consequence of Mendelian sampling and linkage. Genetics Research. 2011;93(1):47–64. 10.1017/S0016672310000480 21226974 PMC3070763

[10] Hill WG, White IMS. Identification of Pedigree Relationship from Genome Sharing. G3: Genes, Genomes, Genetics. 2013;3(9):1553–1571. 10.1534/g3.113.007500 23893739 PMC3755916

[11] Henn BM, Hon L, Macpherson JM, Eriksson N, Saxonov S, Pe’er I, et al Cryptic Distant Relatives Are Common in Both Isolated and Cosmopolitan Genetic Samples. PLOS ONE. 2012;7(4):e34267 10.1371/journal.pone.0034267 22509285 PMC3317976

[12] Ball CA, Barber MJ, Byrnes J, Carbonetto P, Chahine KG, Curtis RE, et al. AncestryDNA Matching White Paper. AncestryDNA; 2016. https://www.ancestry.com/dna/resource/whitePaper/AncestryDNA-Matching-White-Paper.pdf

[13] Broman KW, Weber JL. Characterization of human crossover interference. The American Journal of Human Genetics. 2000;66(6):1911–1926. 10.1086/302923 10801387 PMC1378063

[14] Housworth E, Stahl F. Crossover interference in humans. The American Journal of Human Genetics. 2003;73(1):188–197. 10.1086/376610 12772089 PMC1180580

[15] Campbell CL, Furlotte NA, Eriksson N, Hinds D, Auton A. Escape from crossover interference increases with maternal age. Nature Communications. 2015;6:6260 10.1038/ncomms7260 25695863 PMC4335350

[16] Bhérer C, Campbell CL, Auton A. Refined genetic maps reveal sexual dimorphism in human meiotic recombination at multiple scales. Nature Communications. 2017;8:14994 10.1038/ncomms14994 28440270 PMC5414043

[17] Kong A, Thorleifsson G, Gudbjartsson DF, Masson G, Sigurdsson A, Jonasdottir A, et al Fine-scale recombination rate differences between sexes, populations and individuals. Nature. 2010;467(7319):1099–1103. 10.1038/nature09525 20981099

[18] Donnelly KP. The probability that related individuals share some section of genome identical by descent. Theoretical Population Biology. 1983;23(1):34–63. 10.1016/0040-5809(83)90004-7 6857549

[19] Renwick JH. The mapping of human chromosomes. Annual Review of Genetics. 1971;5(1):81–120. 10.1146/annurev.ge.05.120171.000501 16097652

[20] The International HapMap Consortium. A second generation human haplotype map of over 3.1 million SNPs. Nature. 2007;449(7164):851–861. 10.1038/nature06258 17943122 PMC2689609

[21] Hinch AG, Tandon A, Patterson N, Song Y, Rohland N, Palmer CD, et al The landscape of recombination in African Americans. Nature. 2011;476:170–175. 10.1038/nature10336 21775986 PMC3154982

[22] Ottolini CS, Newnham LJ, Capalbo A, Natesan SA, Joshi HA, Cimadomo D, et al Genome-wide maps of recombination and chromosome segregation in human oocytes and embryos show selection for maternal recombination rates. Nature Genetics. 2015;47:727–735. 10.1038/ng.3306 25985139 PMC4770575

[23] Hou Y, Fan W, Yan L, Li R, Lian Y, Huang J, et al Genome Analyses of Single Human Oocytes. Cell. 2013;155(7):1492–1506. 10.1016/j.cell.2013.11.040 24360273

[24] Wang J, Fan HC, Behr B, Quake SR. Genome-wide Single-Cell Analysis of Recombination Activity and De Novo Mutation Rates in Human Sperm. Cell. 2012;150(2):402–412. 10.1016/j.cell.2012.06.030 22817899 PMC3525523

[25] Lu S, Zong C, Fan W, Yang M, Li J, Chapman AR, et al Probing Meiotic Recombination and Aneuploidy of Single Sperm Cells by Whole-Genome Sequencing. Science. 2012;338(6114):1627–1630. 10.1126/science.1229112 23258895 PMC3590491

[26] Kirkness EF, Grindberg RV, Yee-Greenbaum J, Marshall CR, Scherer SW, Lasken RS, et al Sequencing of isolated sperm cells for direct haplotyping of a human genome. Genome Research. 2013;23(5):826–832. 10.1101/gr.144600.112 23282328 PMC3638138

[27] Bell AD, Mello CJ, Nemesh J, Brumbaugh SA, Wysoker A, McCarroll SA. Insights about variation in meiosis from 31,228 human sperm genomes. bioRxiv. 2019. 10.1038/s41586-020-2347-0 PMC7351608 32494014

[28] Sturtevant AH. The linear arrangement of six sex-linked factors in Drosophila, as shown by their mode of association. Journal of Experimental Zoology. 1913;14(1):43–59. 10.1002/jez.1400140104

[29] Foss E, Lande R, Stahl FW, Steinberg CM. Chiasma interference as a function of genetic distance. Genetics. 1993;133(3):681–691. 8454209 10.1093/genetics/133.3.681 PMC1205352

[30] Zhao H, Speed TP, McPeek MS. Statistical analysis of crossover interference using the chi-square model. Genetics. 1995;139(2):1045–1056. 7713407 10.1093/genetics/139.2.1045 PMC1206355

[31] Vigeland MD. IBDsim: Simulation of Chromosomal Regions Shared by Family Members; 2019. Available from: https://CRAN.R-project.org/package=IBDsim.

[32] Mitchell BD, Kammerer CM, Blangero J, Mahaney MC, Rainwater DL, Dyke B, et al Genetic and environmental contributions to cardiovascular risk factors in Mexican Americans. Circulation. 1996;94(9):2159–2170. 10.1161/01.cir.94.9.2159 8901667

[33] Duggirala R, Blangero J, Almasy L, Dyer TD, Williams KL, Leach RJ, et al Linkage of type 2 diabetes mellitus and of age at onset to a genetic location on chromosome 10q in Mexican Americans. American Journal of Human Genetics. 1999;64(4):1127–1140. 10.1086/302316 10090898 PMC1377837

[34] Hunt KJ, Lehman DM, Arya R, Fowler S, Leach RJ, Göring HH, et al Genome-Wide Linkage Analyses of Type 2 Diabetes in Mexican Americans. Diabetes. 2005;54(9):2655–2662. 10.2337/diabetes.54.9.2655 16123354

[35] Hemani G, Yang J, Vinkhuyzen A, Powell JE, Willemsen G, Hottenga JJ, et al Inference of the Genetic Architecture Underlying BMI and Height with the Use of 20,240 Sibling Pairs. The American Journal of Human Genetics. 2013;93(5):865–875. 10.1016/j.ajhg.2013.10.005 24183453 PMC3965855

[36] Ralph P, Coop G. The Geography of Recent Genetic Ancestry across Europe. PLOS Biology. 2013;11(5):e1001555 10.1371/journal.pbio.1001555 23667324 PMC3646727

[37] Browning BL, Browning SR. Improving the Accuracy and Efficiency of Identity-by-Descent Detection in Population Data. Genetics. 2013;194(2):459–471. 10.1534/genetics.113.150029 23535385 PMC3664855

[38] Williams AL, Housman D, Rinard M, Gifford D. Rapid haplotype inference for nuclear families. Genome Biology. 2010;11(10):R108 10.1186/gb-2010-11-10-r108 21034477 PMC3218664

[39] Abecasis GR, Cherny SS, Cookson WO, Cardon LR. Merlin—rapid analysis of dense genetic maps using sparse gene flow trees. Nat Genet. 2002;30(1):97–101. 10.1038/ng786 11731797

[40] Browning SR, Browning BL. Haplotype phasing: existing methods and new developments. Nature Reviews Genetics. 2011;12(10):703–714. 10.1038/nrg3054 21921926 PMC3217888

[41] Manichaikul A, Mychaleckyj JC, Rich SS, Daly K, Sale M, Chen WM. Robust relationship inference in genome-wide association studies. Bioinformatics. 2010;26(22):2867–2873. 10.1093/bioinformatics/btq559 20926424 PMC3025716

[42] Karlin S, Taylor HM. A First Course in Stochastic Processes. 2nd ed Academic Press; 1975.

[43] Yakovlev G, Rundle JB, Shcherbakov R, Turcotte DL. Inter-arrival time distribution for the non-homogeneous Poisson process. arXiv. 2005;cond-mat/0507657.

[44] Qiao Y, Sannerud J, Basu-Roy S, Hayward C, Williams AL. Distinguishing pedigree relationships using multi-way identical by descent sharing and sex-specific genetic maps. bioRxiv. 2019. 10.1016/j.ajhg.2020.12.004 PMC7820736 33385324

[45] Gudbjartsson DF, Thorvaldsson T, Kong A, Gunnarsson G, Ingolfsdottir A. Allegro version 2. Nature Genetics. 2005;37(10):1015–1016. 10.1038/ng1005-1015 16195711

[46] Dietter J, Mattheisen M, Fürst R, Rüschendorf F, Wienker TF, Strauch K. Linkage analysis using sex-specific recombination fractions with GENEHUNTER-MODSCORE. Bioinformatics. 2006;23(1):64–70. 10.1093/bioinformatics/btl539 17060360

[47] Fingerlin TE, Abecasis GR, Boehnke M. Using sex-averaged genetic maps in multipoint linkage analysis when identity-by-descent status is incompletely known. Genetic Epidemiology. 2006;30(5):384–396. 10.1002/gepi.20151 16685713

[48] Mukhopadhyay N, Weeks DE. Linkage analysis of adult height with parent-of-origin effects in the Framingham Heart Study. BMC Genetics. 2003;4(1):S76 10.1186/1471-2156-4-S1-S76 14975144 PMC1866515

[49] Browning S. Pedigree Data Analysis With Crossover Interference. Genetics. 2003;164(4):1561–1566. 12930760 10.1093/genetics/164.4.1561 PMC1462645

[50] Thompson EA. MCMC Estimation of Multi-locus Genome Sharing and Multipoint Gene Location Scores. International Statistical Review. 2000;68(1):53–73. 10.1111/j.1751-5823.2000.tb00387.x

[51] Lin S, Speed TP. Relative efficiencies of the Chi-square recombination models for gene mapping with human pedigree data. Annals of Human Genetics. 1999;63(1):81–95. 10.1046/j.1469-1809.1999.6310081.x 10738522

[52] Kong A, Thorleifsson G, Frigge ML, Masson G, Gudbjartsson DF, Villemoes R, et al Common and low-frequency variants associated with genome-wide recombination rate. Nature Genetics. 2013;46:11–16. 10.1038/ng.2833 24270358

[53] The International Multiple Sclerosis Genetics Consortium, The Wellcome Trust Case Control Consortium 2, et al Genetic risk and a primary role for cell-mediated immune mechanisms in multiple sclerosis. Nature. 2011;476(7359):214–219. 10.1038/nature10251 21833088 PMC3182531

[54] Williams AL, Genovese G, Dyer T, Altemose N, Truax K, Jun G, et al Non-crossover gene conversions show strong GC bias and unexpected clustering in humans. eLife. 2015; 4:e04637 10.7554/eLife.04637 PMC4404656 25806687

[55] Ramstetter MD, Dyer TD, Lehman DM, Curran JE, Duggirala R, Blangero J, et al Benchmarking Relatedness Inference Methods with Genome-Wide Data from Thousands of Relatives. Genetics. 2017;207(1):75–82. 10.1534/genetics.117.1122 28739658 PMC5586387

[56] Lander ES, Green P. Construction of multilocus genetic linkage maps in humans. Proceedings of the National Academy of Sciences. 1987;84(8):2363–2367. 10.1073/pnas.84.8.2363 PMC304651 3470801

[57] Seidman DN, Shenoy SA, Kim M, Babu R, Dyer TD, Lehman DM, et al. Rapid, Phase-Free Detection of Long Identical by Descent Segments Enables Fast Relationship Classification. (Under review). 2019. 10.1016/j.ajhg.2020.02.012 PMC7118564 32197076

[58] Gravel S. Population Genetics Models of Local Ancestry. Genetics. 2012;191:607–619. 10.1534/genetics.112.139808 22491189 PMC3374321

[59] Cox DR, Smith WL. On the Superposition of Renewal Processes. Biometrika. 1954;41:91–99. 10.2307/2333008

