# Supplementary figures and images for: Crossover interference and sex-specific genetic maps shape identical by descent sharing in close relatives
Source: PLoS Genet. 2019 Dec 20;15(12):e1007979. doi: 10.1371/journal.pgen.1007979 (PMC6944377; doi:10.1371/journal.pgen.1007979)

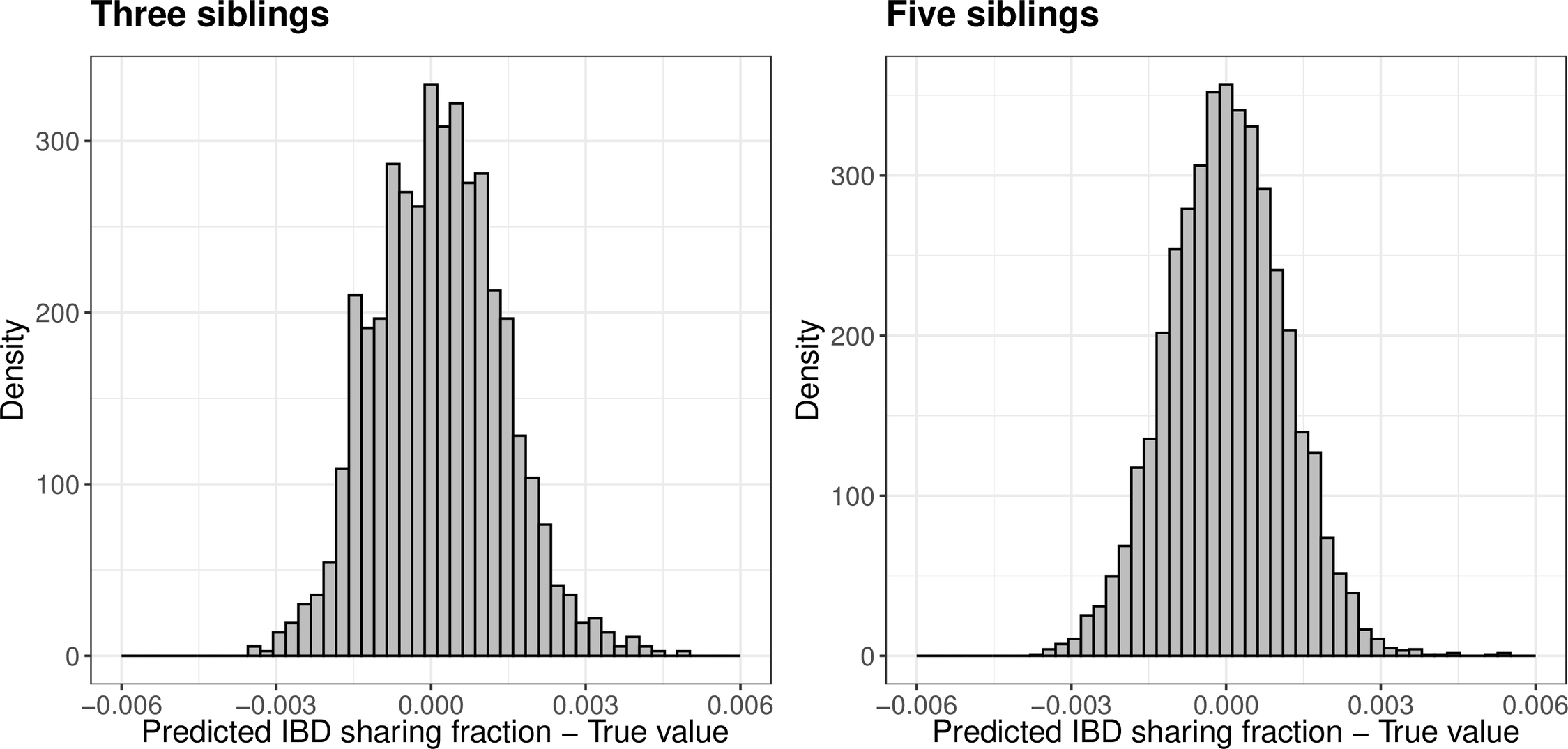

Supplement: S1 Fig — IBD sharing fractions are from the full sibling pairs of 500 simulated nuclear families with three children (left) and 500 with five children (right). We phased these families and extracted IBD sharing estimates as described in Methods. (TIF) [file pgen.1007979.s001.tif]

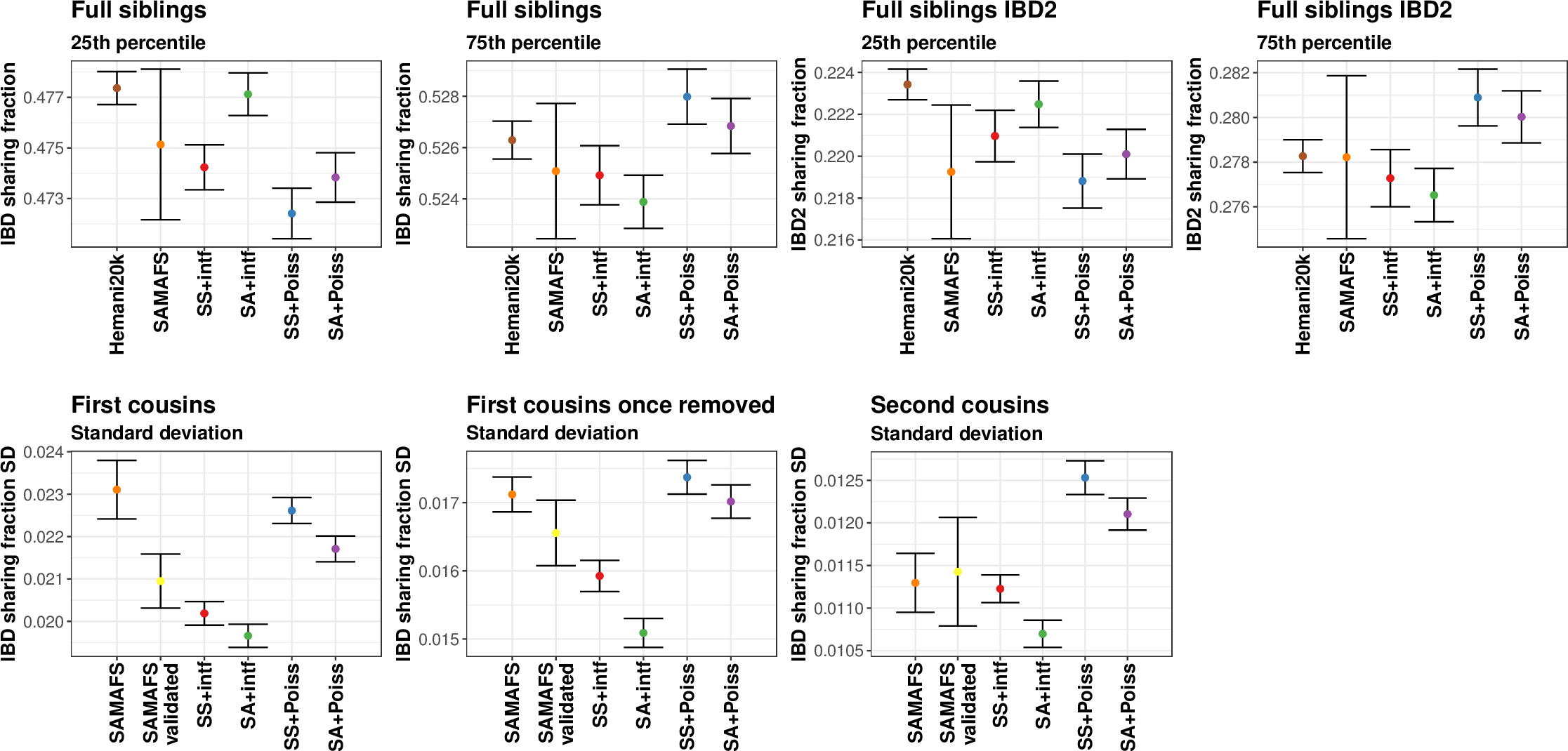

Supplement: S2 Fig — Points are from the SAMAFS, SAMAFS-validated subset (except full siblings), Hemani20k set (only full siblings), and the simulation models. The latter are labeled using abbreviations given in the main text. Bars indicate 95% confidence interval (±1.96 standard errors) as calculated from 1,000 bootstrap samples. SD indicates standard deviation. (TIF) [file pgen.1007979.s002.tif]

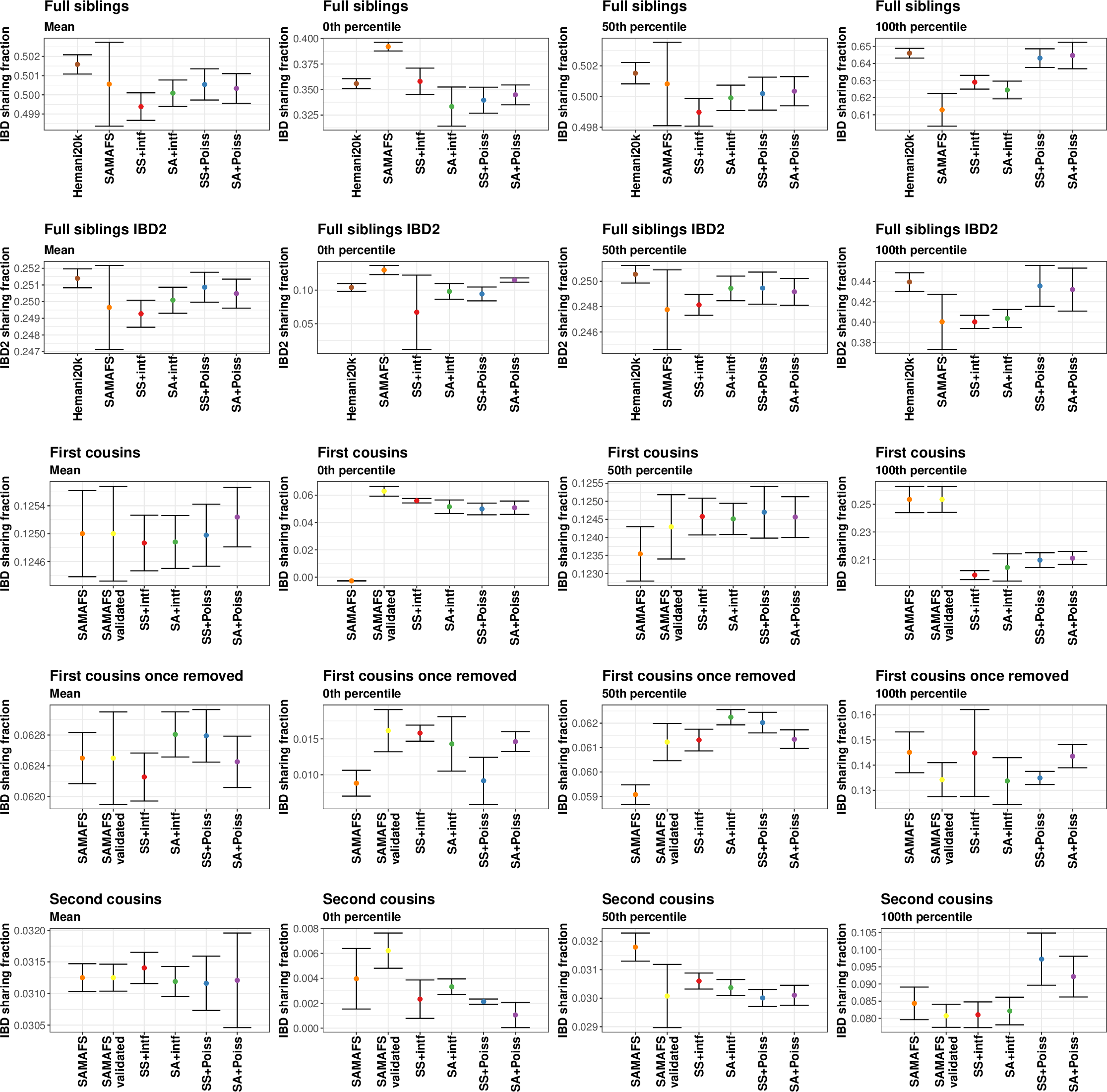

Supplement: S3 Fig — Points are from the SAMAFS, SAMAFS-validated subset (except full siblings), Hemani20k set (only full siblings), and the simulation models. The latter are labeled using abbreviations given in the main text. The SAMAFS and SAMAFS-validated values are mean-shifted to match expectations for the first cousins, first cousins once removed, and second cousins, but are unaltered for the full sibling and the full sibling IBD2 quantities. Bars indicate 95% confidence interval (±1.96 standard errors) as calculated from 1,000 bootstrap samples. (TIF) [file pgen.1007979.s003.tif]

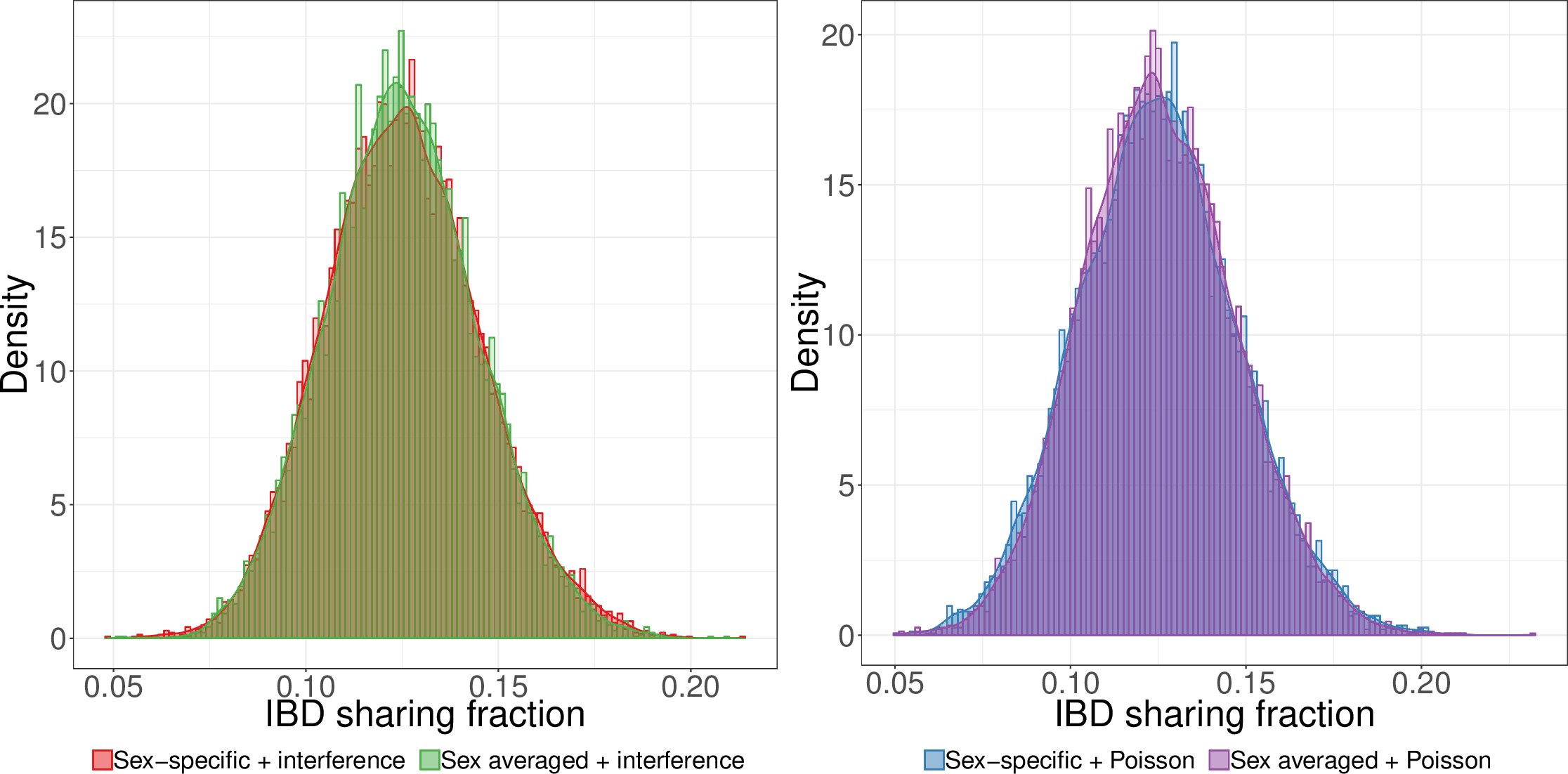

Supplement: S4 Fig — Sex-specific and sex averaged distributions heavily overlap both when using an interference (left) and a Poisson (right) model for inter-crossover distances. (TIF) [file pgen.1007979.s004.tif]

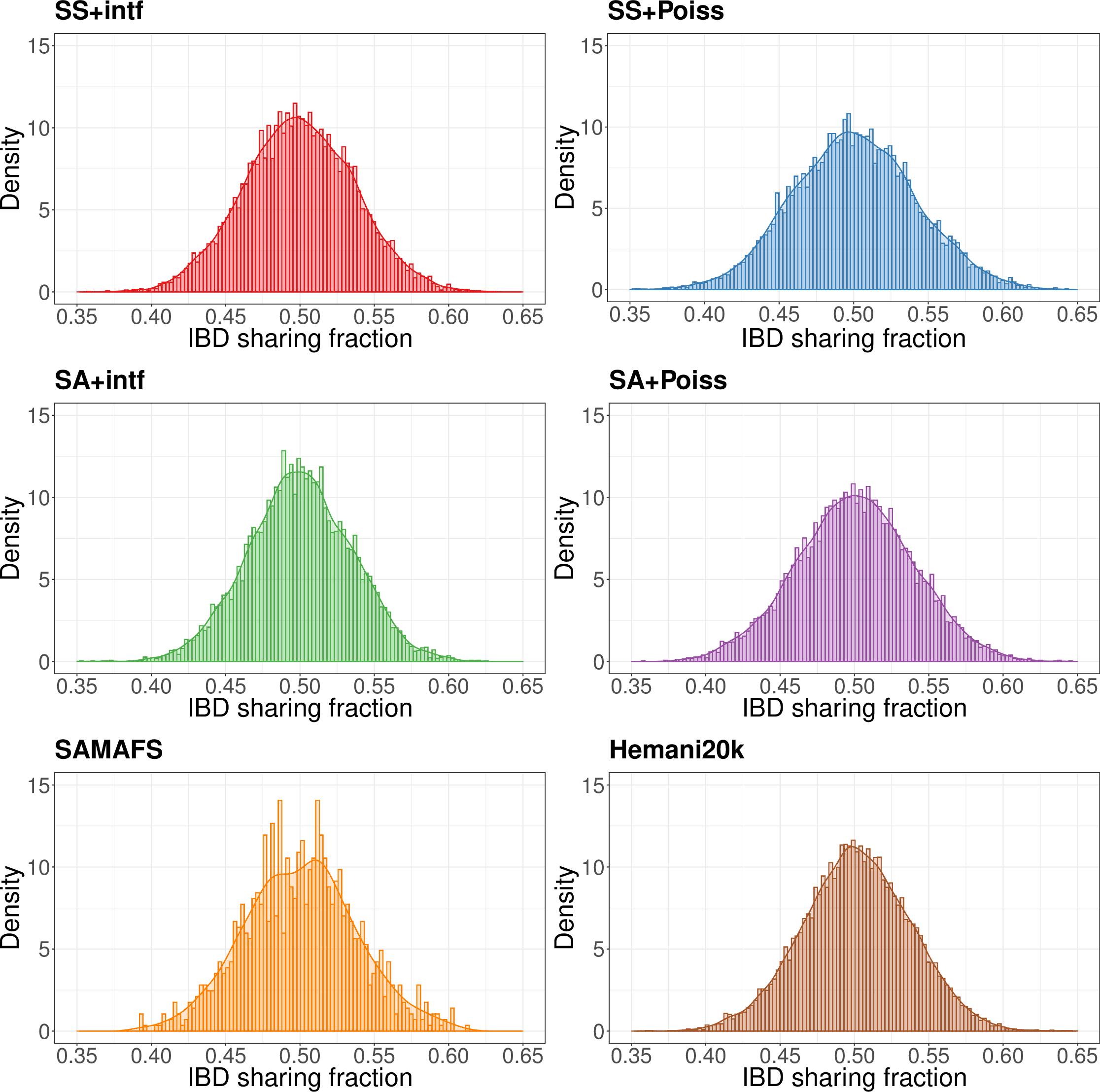

Supplement: S5 Fig — Each simulation includes 10,000 full sibling pairs, the SAMAFS data include 1,128 pairs (Methods), and the Hemani20k data total 20,240 pairs. (TIF) [file pgen.1007979.s005.tif]

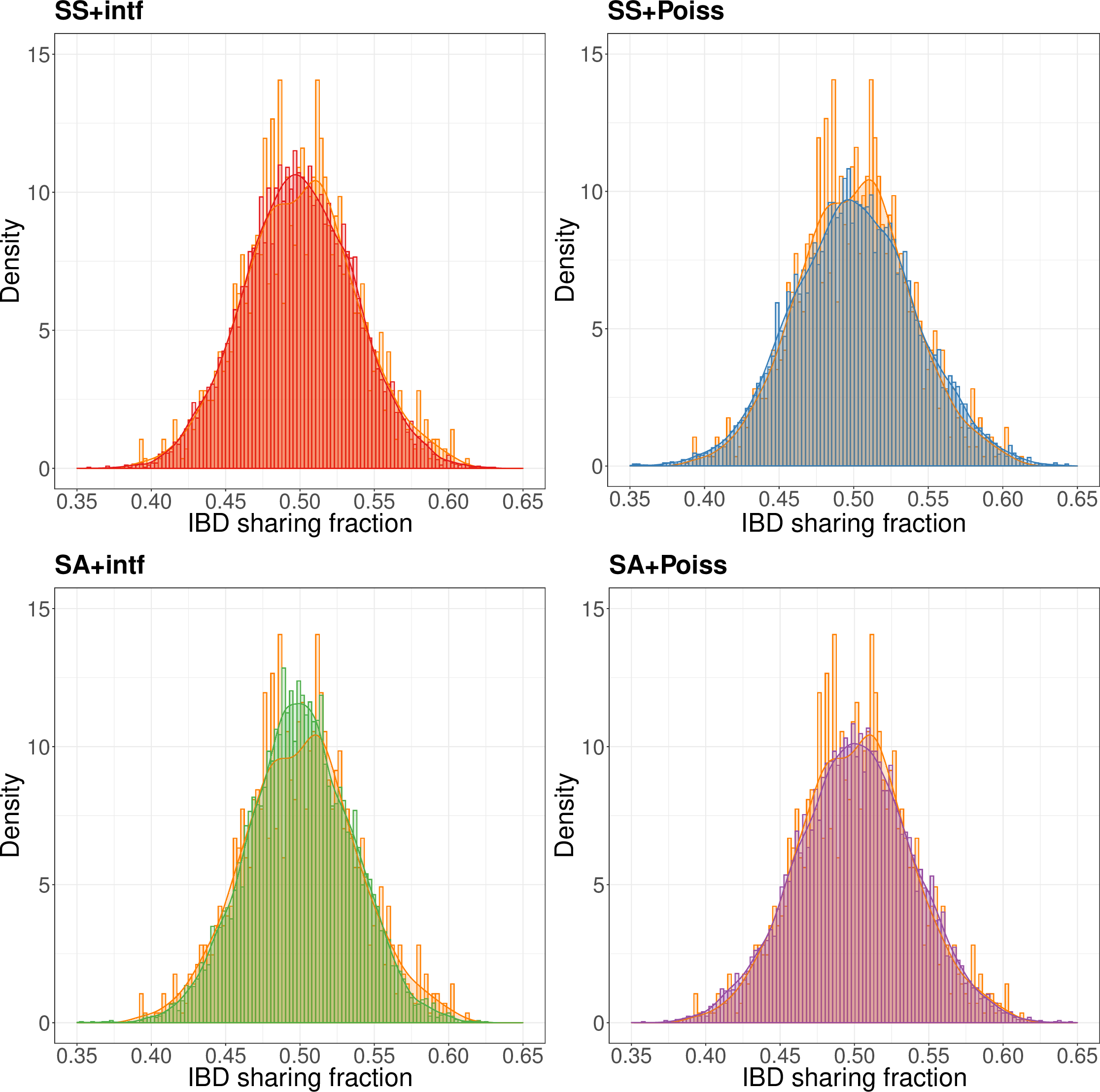

Supplement: S6 Fig — Plots are histograms of the 1,128 SAMAFS pairs and 10,000 simulated pairs generated under each of the crossover models, as indicated. (TIF) [file pgen.1007979.s006.tif]

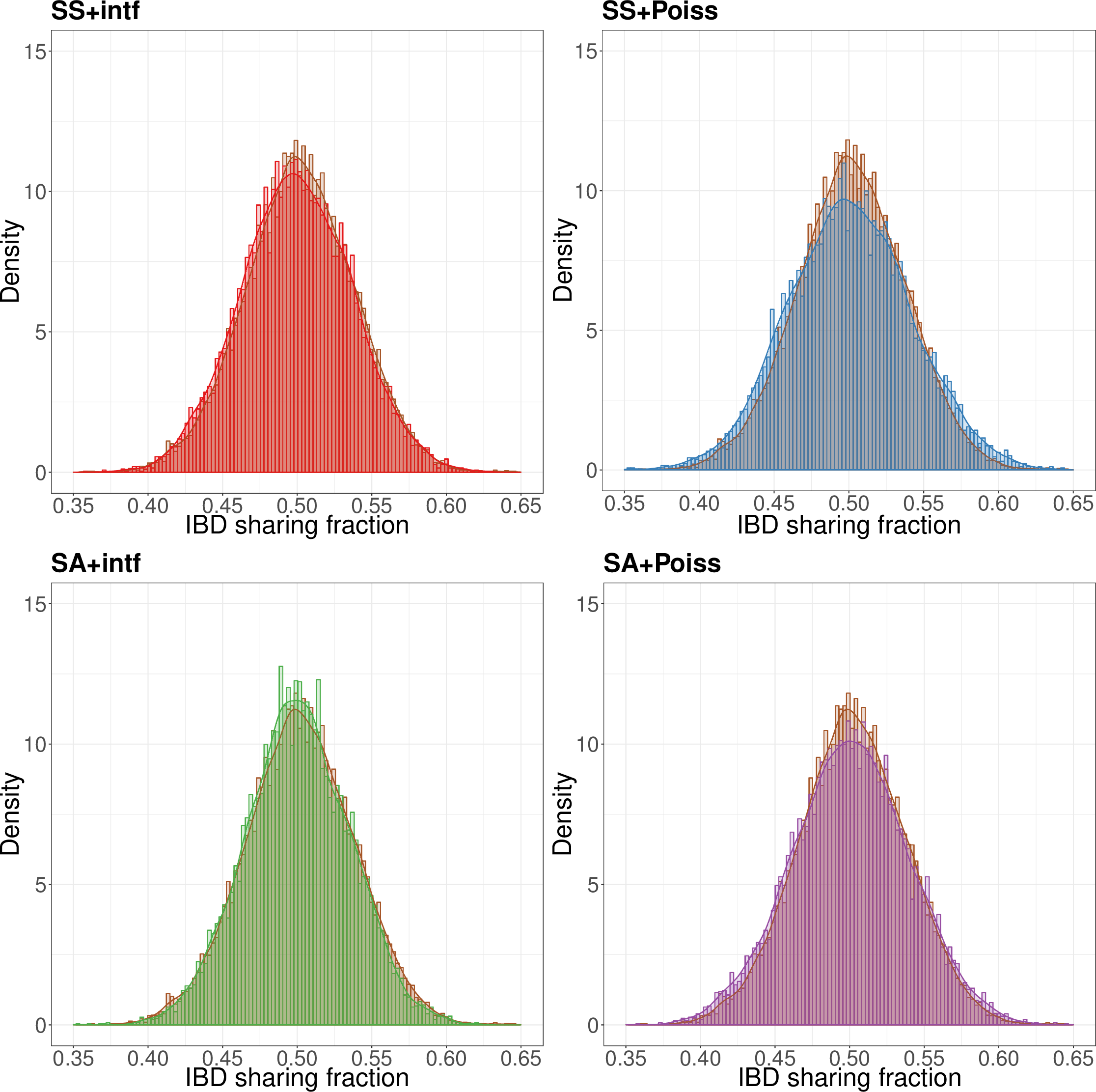

Supplement: S7 Fig — Plots are histograms of the 20,240 Hemani20k pairs and 10,000 simulated pairs generated under each of the crossover models, as indicated. (TIF) [file pgen.1007979.s007.tif]

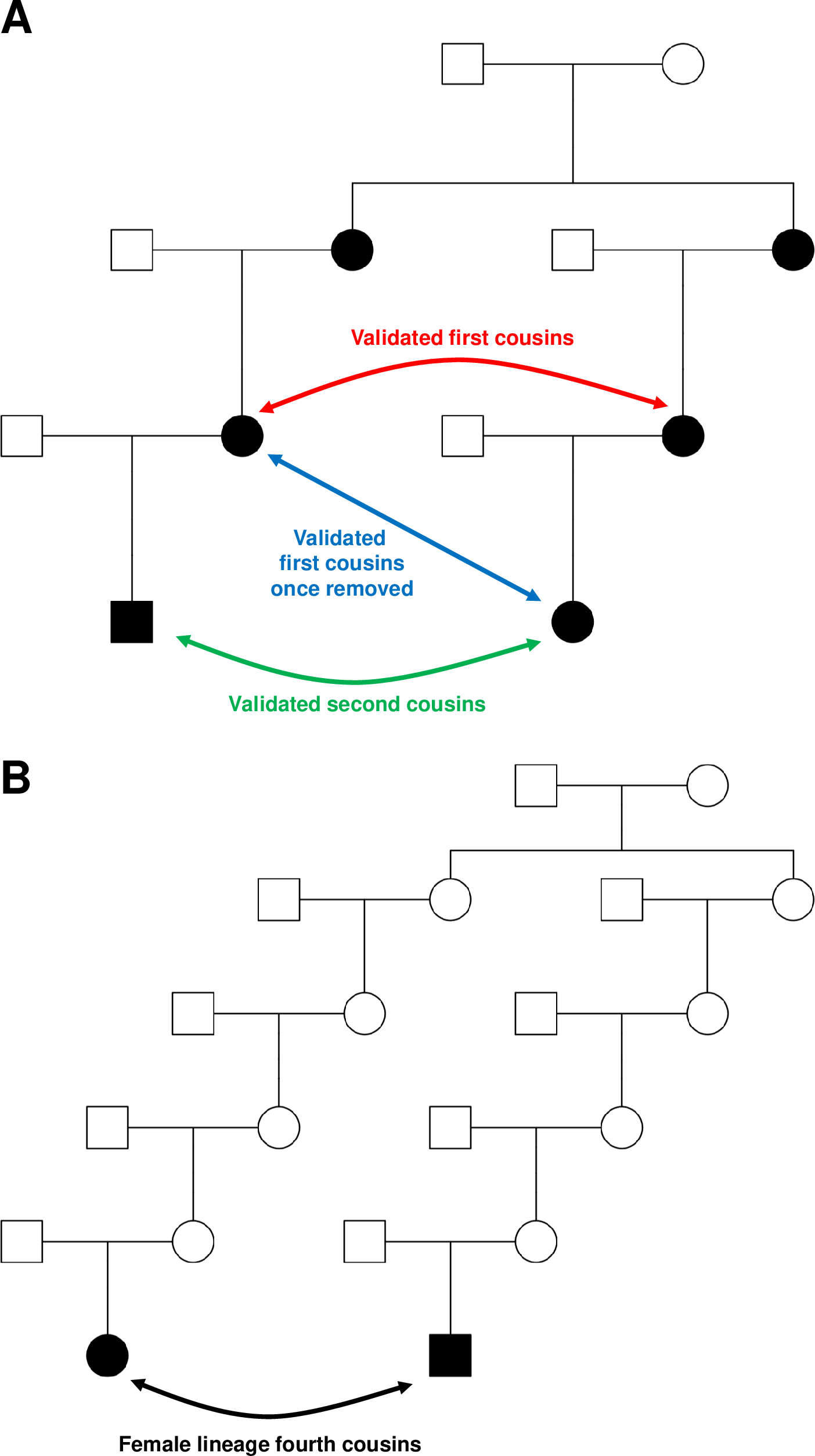

Supplement: S8 Fig — (A) SAMAFS-validated pairs are required to be descended from a genotyped (black) full sibling pair and to have genotyped parent-child relatives that directly connect them to the full siblings. We further require that both the ancestral full sibling pair and all parent-child pairs be inferred as first degree relatives by Refined IBD. (B) Plot of female-lineage fourth cousins. (TIF) [file pgen.1007979.s008.tif]

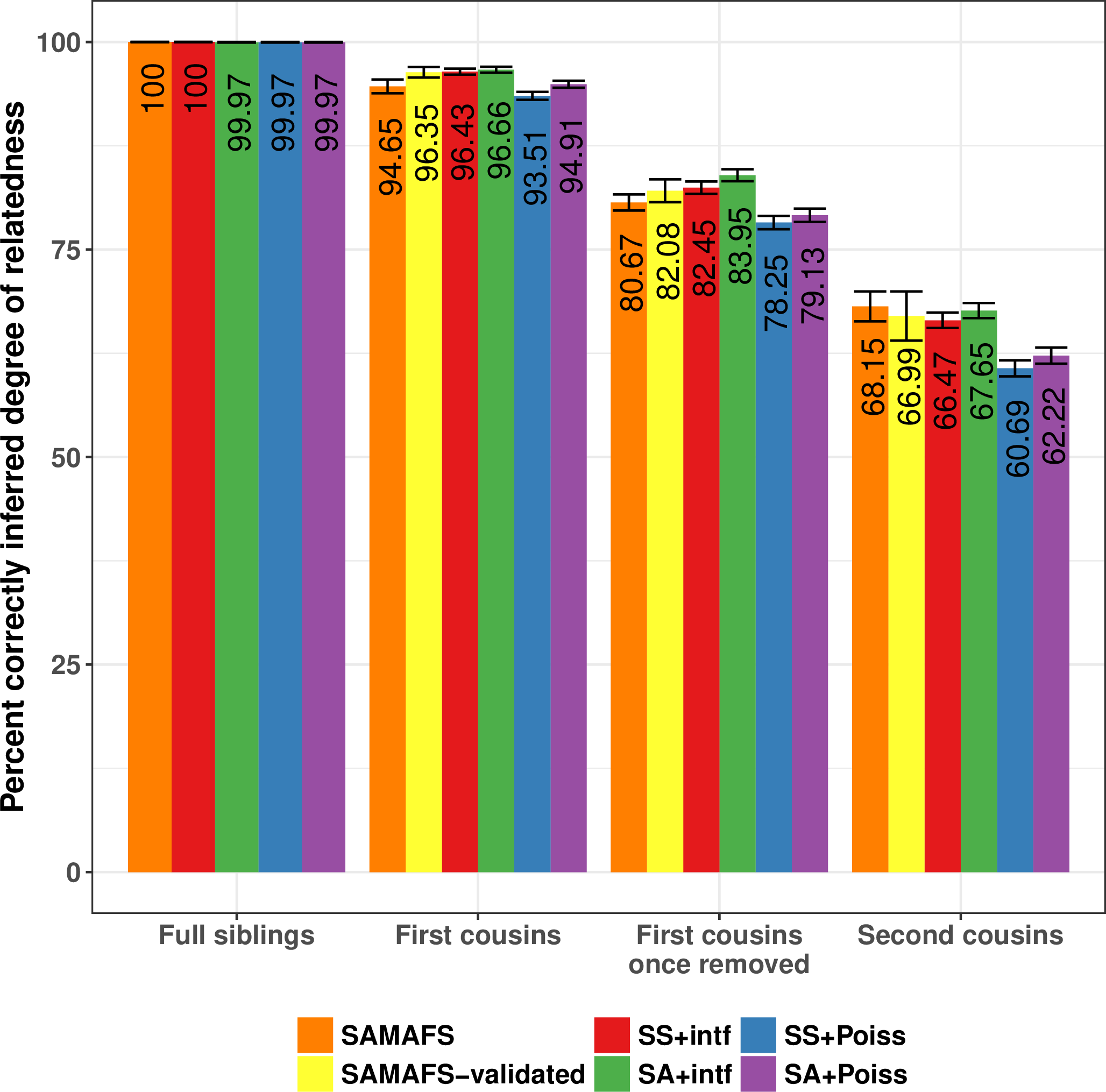

Supplement: S9 Fig — Degrees are inferred from kinship coefficients, with the latter calculated using inferred (for SAMAFS and SAMAFS-validated) or true (for the simulations) IBD segments (see Methods). Bars indicate 95% confidence interval (±1.96 standard errors) based on 1,000 bootstrap samples over relative pairs. (TIF) [file pgen.1007979.s009.tif]

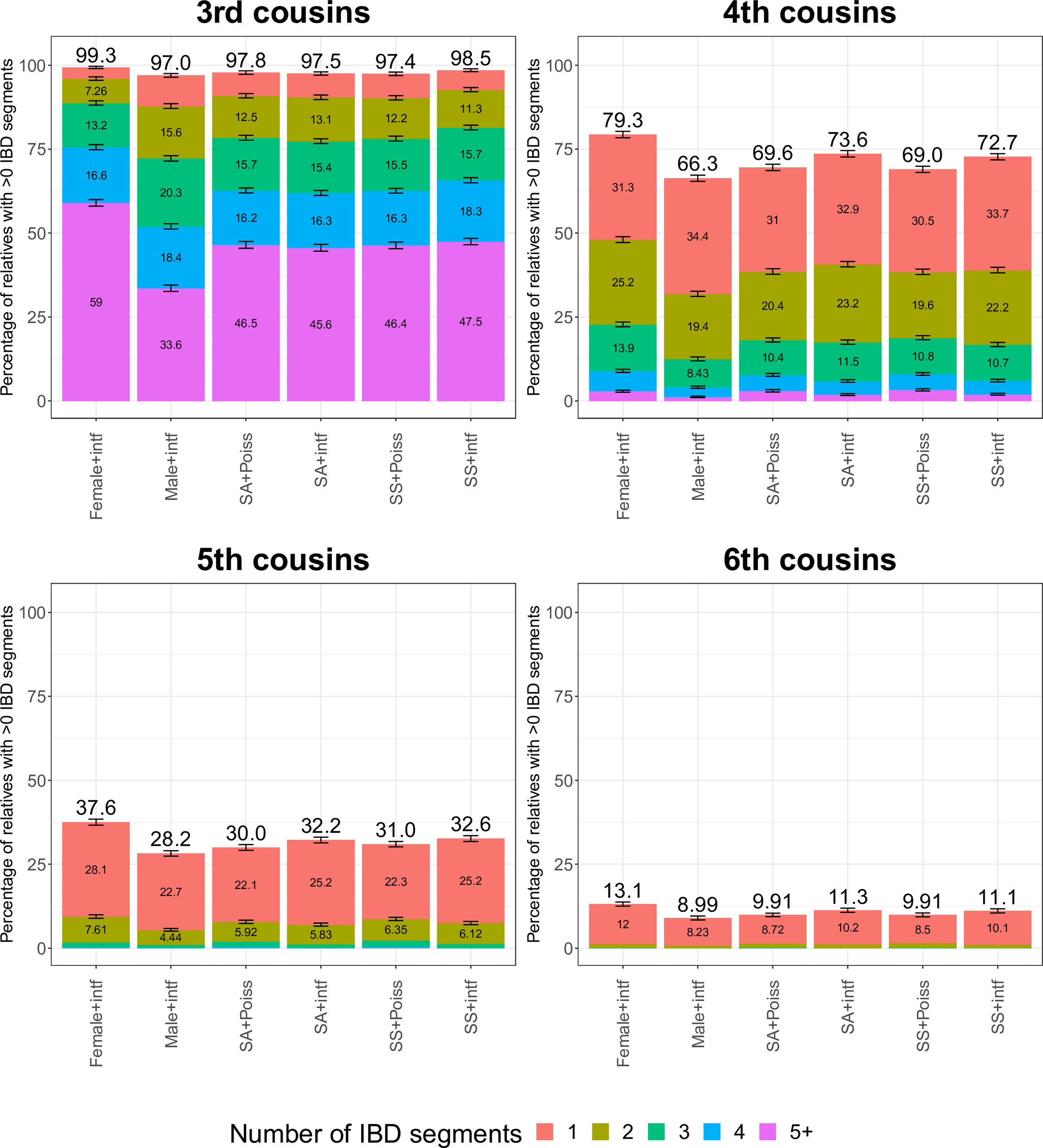

Supplement: S10 Fig — Percentages above each bar indicate the fraction of simulated relatives (of 10,000 for each scenario) that have at least one segment shared. Within stacked colored bars, numbers are the percentage of relatives that share the indicated number of IBD segments. Error bars above a given stacked bar is the 95% confidence interval (±1.96 standard errors) of the percentage of relatives that share the indicated number of segments based on 1,000 bootstrap samples. (TIF) [file pgen.1007979.s010.tif]

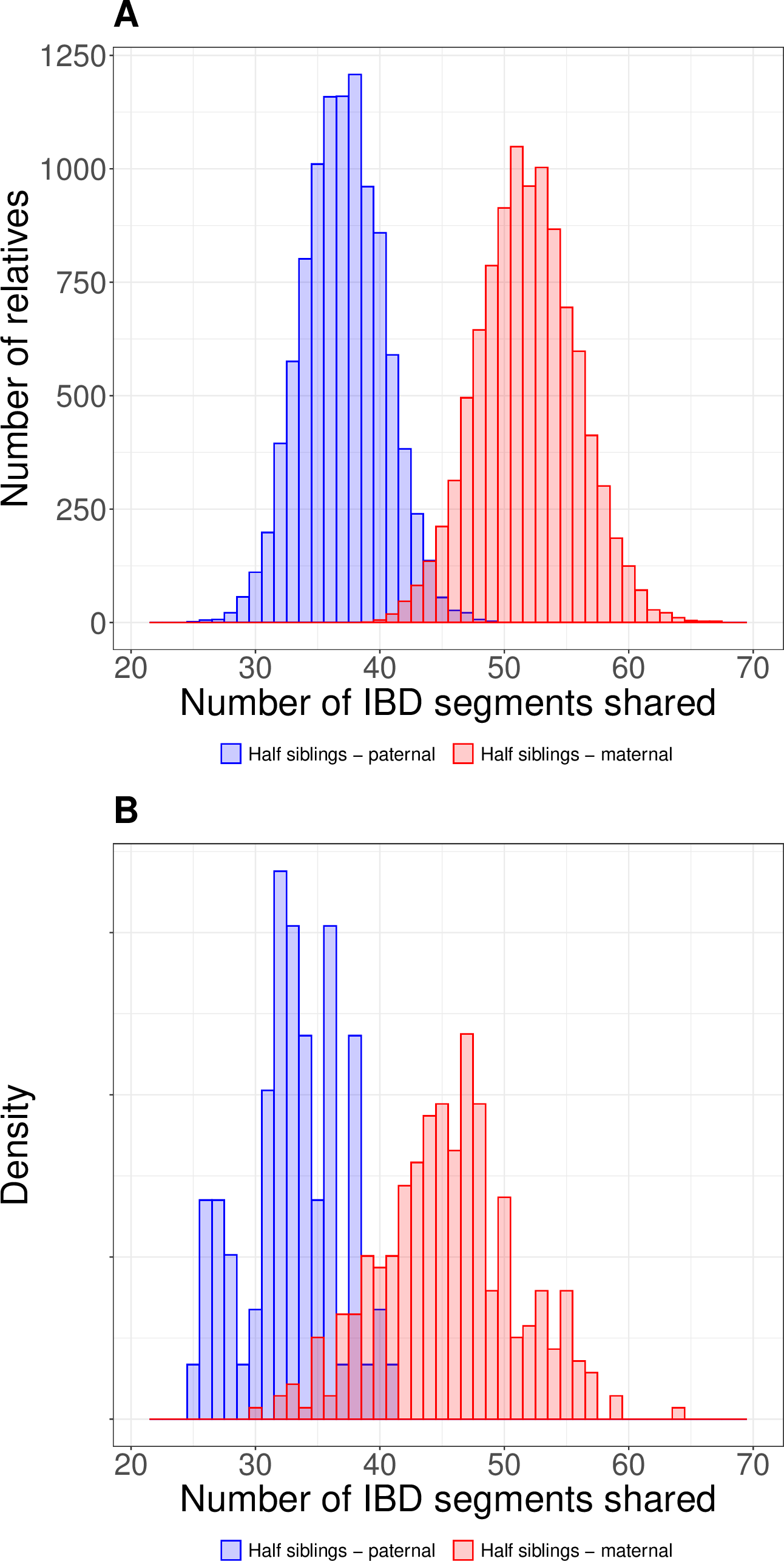

Supplement: S11 Fig — (A) 10,000 simulated pairs for both types of half-siblings under the SS+intf model. (B) Number of IBD segments shared between maternal and paternal half-siblings within SAMAFS. (TIF) [file pgen.1007979.s011.tif]

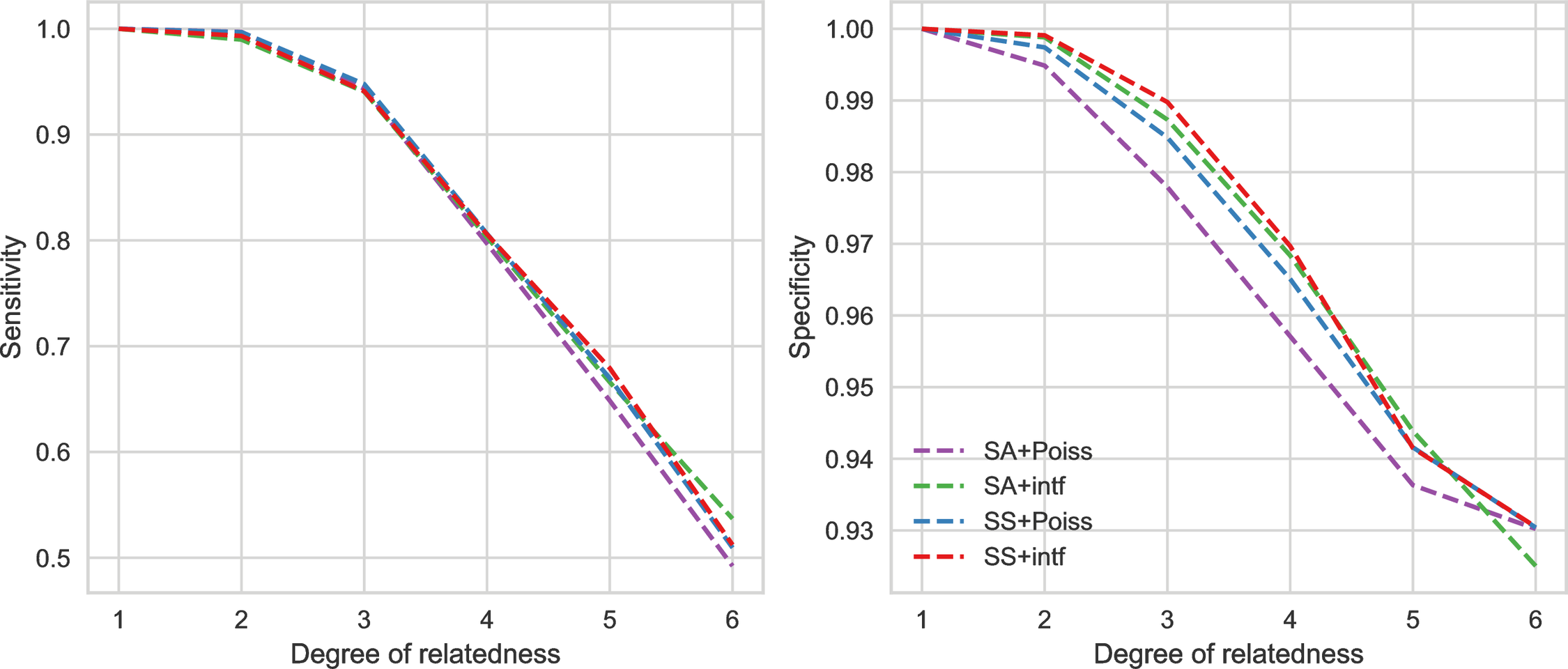

Supplement: S12 Fig — The sensitivity (left) and specificity (right) of the classifiers, with the crossover model used to simulate the training data for each KDE indicated by line color. Rates are from 4,000 pairs of relatives in each degree, each simulated under the SS+intf model. (TIF) [file pgen.1007979.s012.tif]

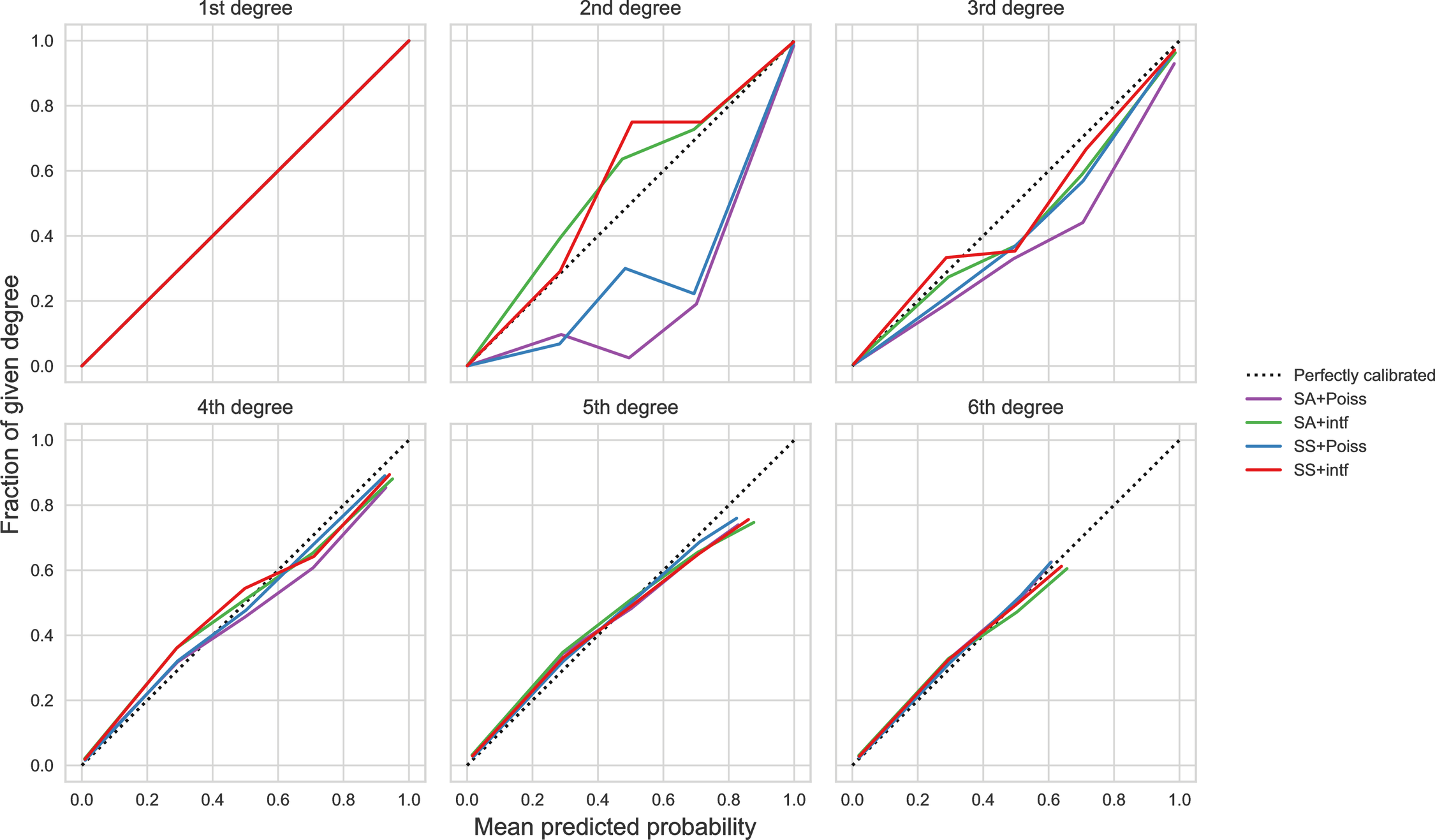

Supplement: S13 Fig — We binned the predicted probabilities into bins of size 0.2. In each plot, the x-axis shows the per-bin mean predicted probability and the y-axis indicates the proportion of pairs that are of the given degree in the corresponding bin. The crossover model used to simulate the training data for each KDE is indicated by line color. (TIF) [file pgen.1007979.s013.tif]

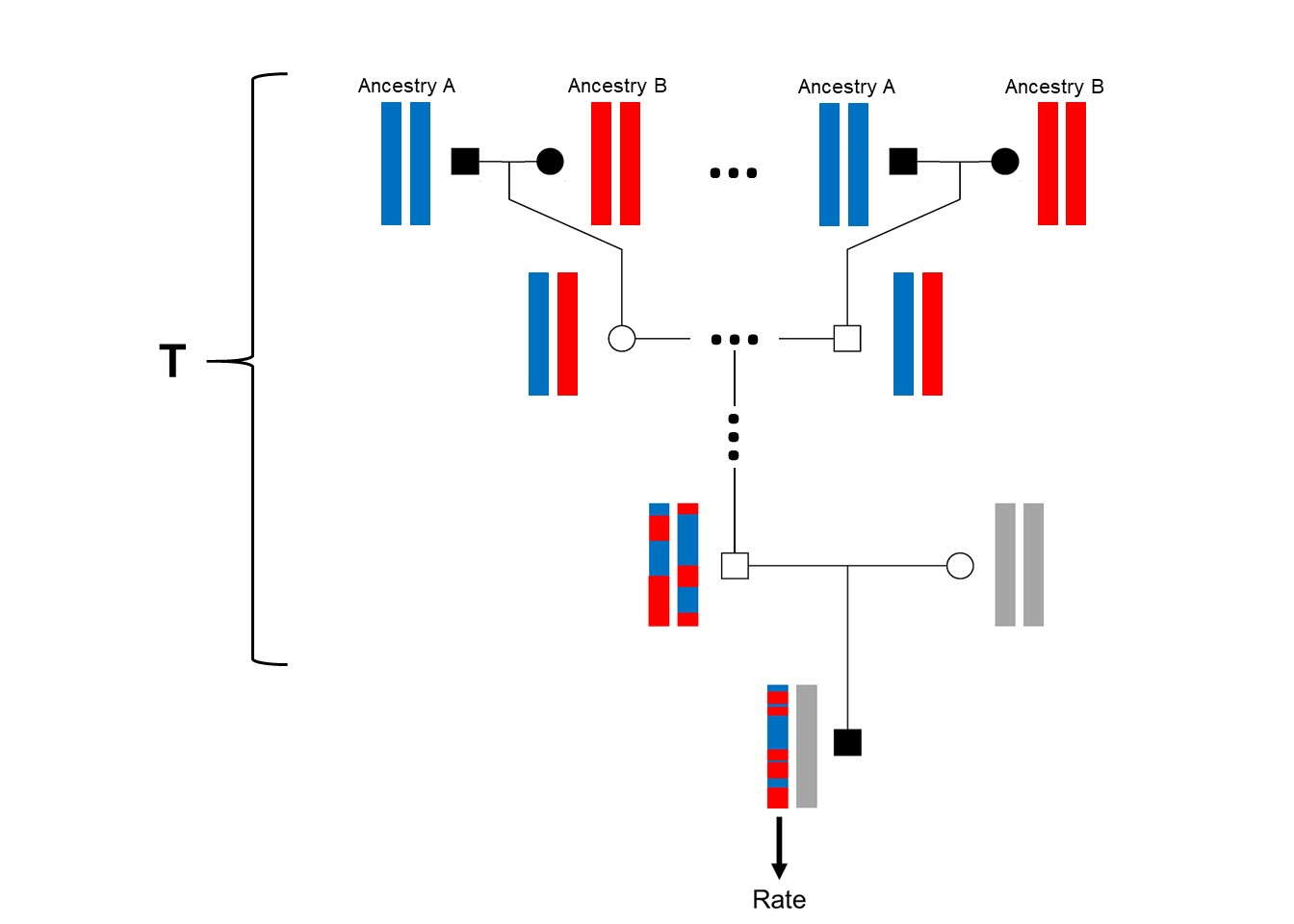

Supplement: S14 Fig — The number of generations since admixture, T, varies, and the number of unadmixed ancestors in the first generation is 2T/2. Plot shows T = 3 (ignoring ellipses) with paternal ancestors. The simulated ancestors are randomly either maternal or paternal. IBD segments between samples with filled shapes define local ancestry regions. (TIF) [file pgen.1007979.s014.tif]

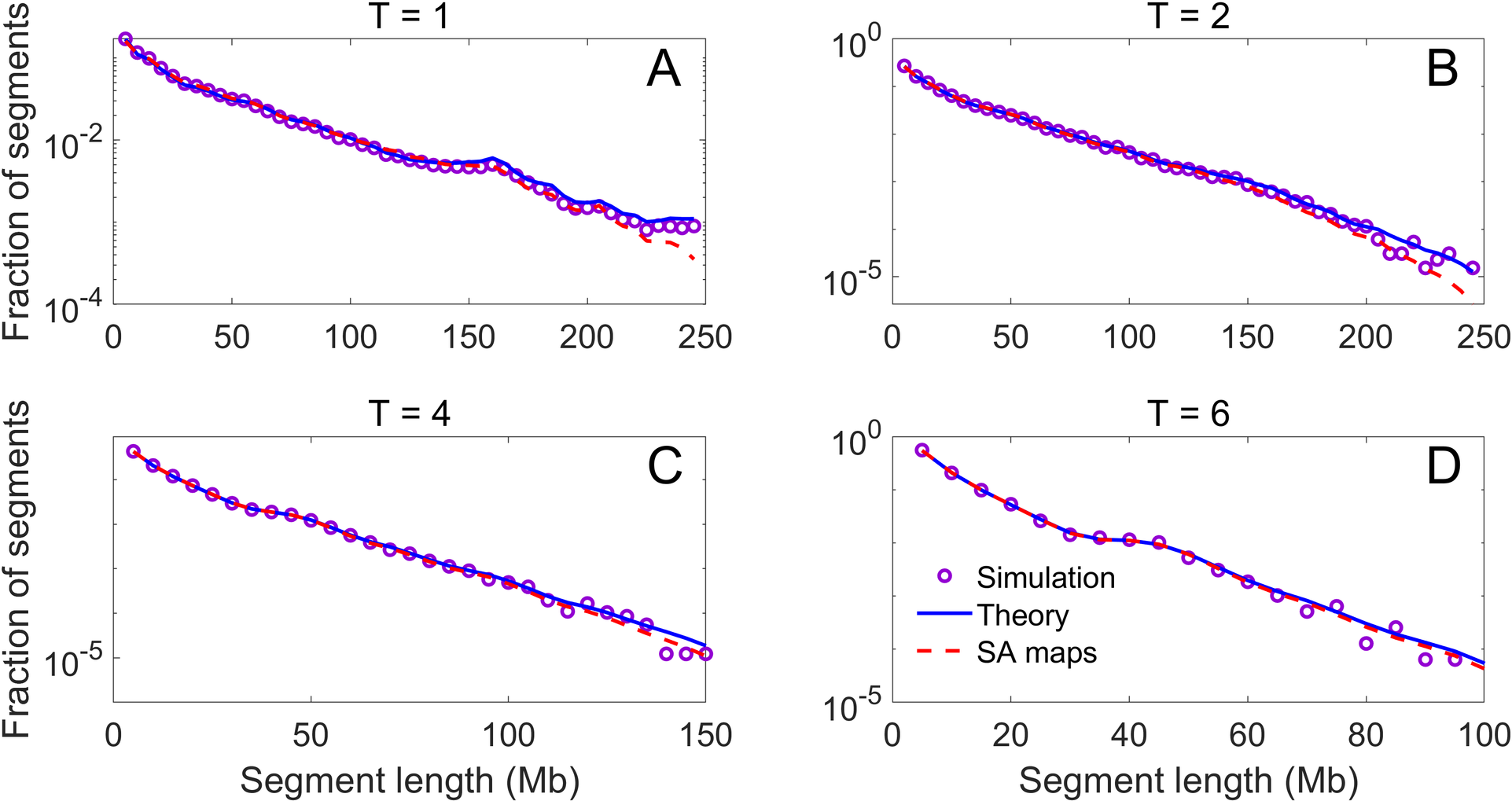

Supplement: S15 Fig — We used Ped-sim to simulate half-cousins with a common ancestor T = 1, 2, 4, 6 generations ago (panels A-D, respectively) under the SS+Poiss model, extracting IBD segment lengths in bp for chromosome 1. Each panel shows the simulated distribution of IBD segment lengths (over 105 pairs for T = 1, 2 and 106 pairs otherwise; purple circles), the theory from Eq (2) (blue lines), and the expectation based on a sex-averaged map (red dashed lines). To evaluate Eq (2) we replaced the integrals with sums over discrete coordinates, evenly separated by 104 bp. (TIF) [file pgen.1007979.s015.tif]

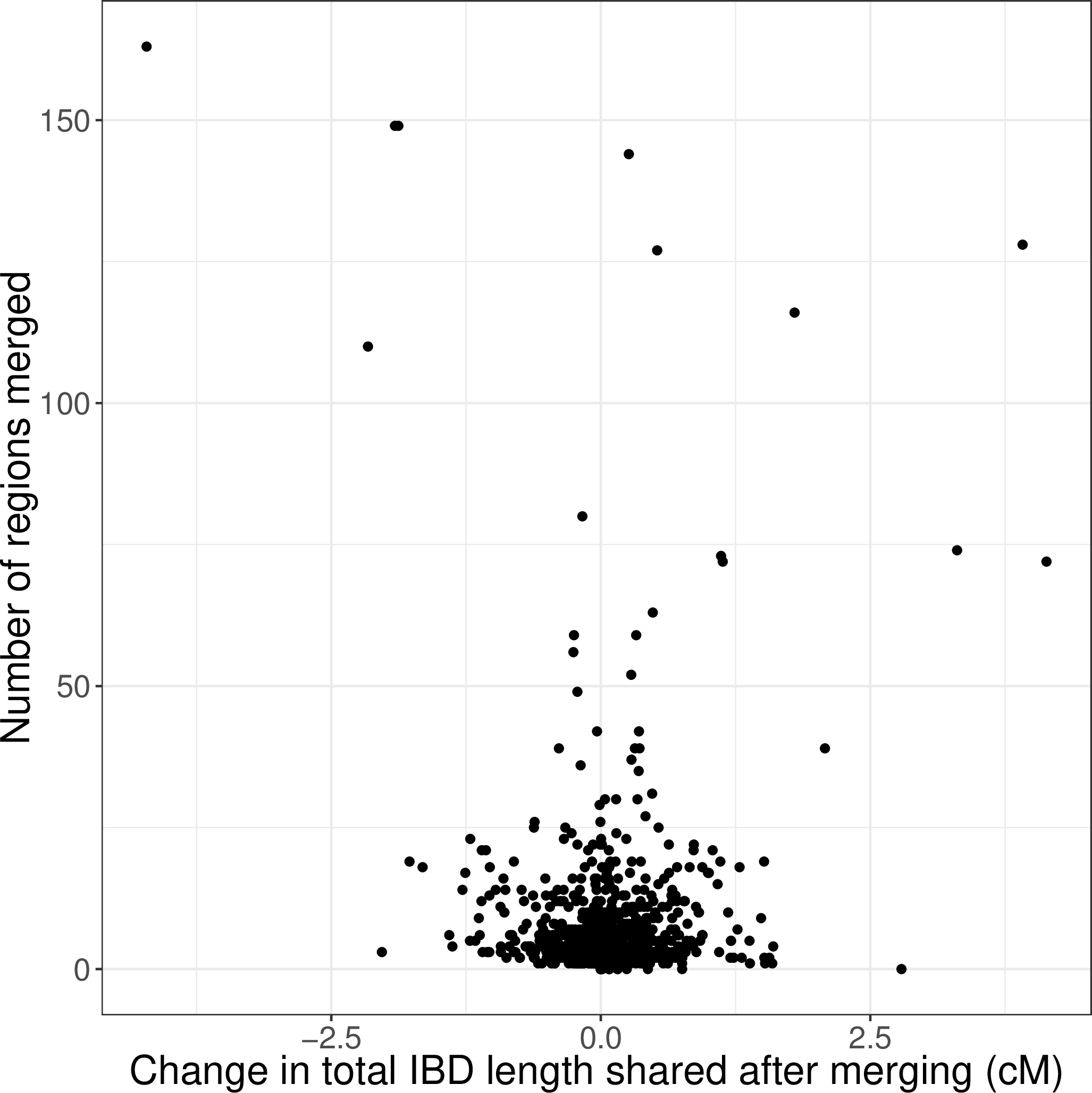

Supplement: S16 Fig — Predicted IBD segments consisting of < 10 informative SNPs are potentially false, and we merged these with the previous segment (Methods). On average, this resulted in a total of 6.75 merged regions per pair across all autosomes, and an average increase of 0.0495 cM shared. No pair gained or lost more than 4.3 cM of IBD regions. (TIF) [file pgen.1007979.s016.tif]
